# Supplementary material for: NHS ‘Learning from Deaths’ reports: a qualitative and quantitative document analysis of the first year of a countrywide patient safety programme
Source: BMJ Open. 2021 Jul 7;11(7):e046619. doi: 10.1136/bmjopen-2020-046619 (PMC8264864; doi:10.1136/bmjopen-2020-046619)
Supplement: Author's manuscript [file bmjopen-2020-046619.draft_revisions.pdf]

BMJ Open is committed to open peer review. As part of this commitment we make the peer review history of every article we publish publicly available.

When an article is published we post the peer reviewers' comments and the authors' responses online. We also post the versions of the paper that were used during peer review. These are the versions that the peer review comments apply to.

The versions of the paper that follow are the versions that were submitted during the peer review process. They are not the versions of record or the final published versions. They should not be cited or distributed as the published version of this manuscript.

BMJ Open is an open access journal and the full, final, typeset and author-corrected version of record of the manuscript is available on our site with no access controls, subscription charges or pay-per-view fees (http://bmjopen.bmj.com).

If you have any questions on BMJ Open's open peer review process please email info.bmjopen@bmj.com

BMJ Open

NHS Hospital 'Learning from Deaths' reports: A qualitative and quantitative analysis of the first year of a countrywide patient safety programme

Journal:	*BMJ Open*
Manuscript ID	bmjopen-2020-046619
Article Type:	Original research
Date Submitted by the Author:	10-Nov-2020
Complete List of Authors:	Brummell, Zoe; University College London Hospitals NHS Foundation Trust, Anaesthetics Department<br>Vindrola-Padros, Cecilia; University College London, Department of Targeted Intervention; UCL, UCL<br>Braun, Dorit; Advisor/Lived experience<br>Moonesinghe, S. Ramani; UCL/UCLH Surgical Outcomes Research Centre, Centre for Perioperative Medicine, Department for Targeted Intervention, Division of Surgery and Interventional Science; Health Services Research Centre, , National Institute for Academic Anaesthesia, Royal College of Anaesthetists
Keywords:	Health & safety < HEALTH SERVICES ADMINISTRATION & MANAGEMENT, Health policy < HEALTH SERVICES ADMINISTRATION & MANAGEMENT, Organisational development < HEALTH SERVICES ADMINISTRATION & MANAGEMENT, Quality in health care < HEALTH SERVICES ADMINISTRATION & MANAGEMENT, QUALITATIVE RESEARCH

SCHOLARONE™
Manuscripts

I, the Submitting Author has the right to grant and does grant on behalf of all authors of the Work (as defined in the below author licence), an exclusive licence and/or a non-exclusive licence for contributions from authors who are: i) UK Crown employees; ii) where BMJ has agreed a CC-BY licence shall apply, and/or iii) in accordance with the terms applicable for US Federal Government officers or employees acting as part of their official duties; on a worldwide, perpetual, irrevocable, royalty-free basis to BMJ Publishing Group Ltd ("BMJ") its licensees and where the relevant Journal is co-owned by BMJ to the co-owners of the Journal, to publish the Work in this journal and any other BMJ products and to exploit all rights, as set out in our licence.

The Submitting Author accepts and understands that any supply made under these terms is made by BMJ to the Submitting Author unless you are acting as an employee on behalf of your employer or a postgraduate student of an affiliated institution which is paying any applicable article publishing charge ("APC") for Open Access articles. Where the Submitting Author wishes to make the Work available on an Open Access basis (and intends to pay the relevant APC), the terms of reuse of such Open Access shall be governed by a Creative Commons licence – details of these licences and which Creative Commons licence will apply to this Work are set out in our licence referred to above.

Other than as permitted in any relevant BMJ Author's Self Archiving Policies, I confirm this Work has not been accepted for publication elsewhere, is not being considered for publication elsewhere and does not duplicate material already published. I confirm all authors consent to publication of this Work and authorise the granting of this licence.

For peer review only

NHS Hospital 'Learning from Deaths' reports: A qualitative and quantitative analysis of the first year of a countrywide patient safety programme

Authors:

Zoe Brummell (0000-0002-6296-5049), Researcher/Clinician, Department of Anaesthesia, University College London Hospitals NHS Foundation Trust, London, NW1 2BU

Cecilia Vindrola-Padros (0000-0001-7859-1646), Senior Researcher, Department of Targeted Intervention, University College London, Charles Bell House, 43-45 Foley Street, London, W1W 7TY

Dorit Braun, Advisor/Lived experience, Learning from Deaths: Learning and Action Public and Relatives Steering Group member

S. Ramani Moonesinghe (0000-0002-6730-5824), Professor, Centre for Perioperative Medicine, Research Department for Targeted Intervention, University College London Division of Surgery and Interventional Science, Charles Bell House, 43 Foley Street, London, W1W 7TS

Corresponding author:

Zoe Brummell (0000-0002-6296-5049), Researcher/Clinician, Department of Anaesthesia, University College London Hospitals NHS Foundation Trust, London, England

z.brummell@nhs.net

For peer review only

ABSTRACT

Objectives
To review how organisations are using the Learning from Deaths (LfDs) programme to learn from and prevent, potentially preventable deaths.

Introduction
Potentially preventable deaths occur worldwide within healthcare organisations. In England, inconsistencies in how NHS secondary care trusts reviewed, investigated and shared learning from deaths, resulted in the introduction of national guidance on 'Learning from Deaths' in 2017. This guidance provides a 'framework for identifying, reporting, investigating and learning from deaths'. Amendments to NHS Quality Account regulations, legally require NHS trusts in England to report quantitative and qualitative information relating to patient deaths annually. The programme intended trusts would share this learning and take measurable action to prevent future deaths.

Method
We undertook qualitative and quantitative secondary data analysis of all NHS secondary care trust LfDs reports within their 2017/18 Quality Accounts (n=222).

Results
All statutory elements of LfDs reporting were reported by 98 out of 222 (44%) trusts. The percentage of deaths judged more likely than not due to problems in healthcare was between 0% and 13%. The majority of trusts (89%) reported lessons learnt; the most common learning theme was poor communication. 106 out of 222 trusts (48%) have shared or plan to share the learning within their own organisation. The majority of trusts (86%) reported actions taken and 47% discussed or had a plan for assessment of impact. 37 out of 222 trusts (17%) mentioned involvement of bereaved families.

Conclusions
The wide variation in reporting demonstrates that some trusts have engaged fully with LfDs, while other trusts appear to have disengaged with the programme. This may reveal a disparity in organisational learning and patient safety culture which could result in inequity for bereaved families. Many themes identified from the LfD reports have previously been identified by national and international reports and inquiries.

For peer review only

STRENGTHS AND LIMITATIONS OF THIS STUDY

This is the first study to our knowledge to analyse LfD reporting.

All NHS secondary care trusts in England are included in the analysis.

This study has ensured the inclusion of views from bereaved relatives, through PPI involvement. The authors conclude that these views are essential to improving patient safety.

This is an analysis of the very first year of LfDs reporting, many trusts may not have got completely to grips with it yet.

It is important to understand that trusts may be undertaking elements that were not statutory reporting requirements and raises the question of whether public reporting is fully reflective of trust engagement with the LfDs programme.

For peer review only

INTRODUCTION

Globally, adverse events while receiving medical treatment are a leading cause of morbidity and mortality.[1] From studies within the United States and Europe the percentage of ‘preventable’ or ‘potentially preventable’ deaths is likely to lie somewhere between 0.5% and 8.4% of hospital deaths.[2-6] In England between April 2017 and end of March 2018 there were 299,000 deaths occurring in hospital or within 30 days of discharge.[7] This amounts to an estimate of between 1,495 and 25,116 potentially preventable deaths in England in 2017/2018. There is a moral imperative for healthcare organisations to learn from these deaths and take measurable action to prevent potentially preventable deaths. Healthcare organisations are made up of individuals who have the ability to learn: however organisational learning is ‘more than the sum of individual learning’ and is distinct from unreflective action taking.[8] It is more than simply creating change for change’s sake, as an ‘illusion of learning’.[9] Organisational learning is the ability to apply knowledge and understanding to increase effective organisational action.[8, 10] Effective organisational learning is crucial to improve patient safety and probably requires both safety-I (understanding why things go wrong) and safety-II (understanding why things go right) approaches.[11, 12] In addition central regulation and performance management may have some effect on improving care, but quality improvement, leadership, public engagement, proper resourcing, education, and training are needed for a safer health service.[12]

In April 2016 an independent review demonstrated a lack of systematic approach and meaningful change in response to unexpected deaths at Southern Health NHS Foundation Trust.[13] The Care Quality Commission (CQC), which is responsible for monitoring, inspection and regulation of healthcare services within England, conducted a wider review into the investigations of deaths. They found inconsistencies in the way NHS trusts became aware of, investigated and shared learning from deaths.[14] In response, the NHS launched a new programme of work to improve standards. This included national guidance on Learning from Deaths’ (LfDs), providing a ‘framework for NHS Trusts on identifying, reporting, investigating and learning from deaths in care’. The objectives of the guidance included supporting the NHS in England to develop an understanding of why deaths contributed to by problems in care happen, with the aim of ensuring that findings are shared and acted upon, to prevent recurrence.[15] In July 2017 guidance was published on implementing the LfDs framework at trust board level,[16] and amendments to statutory regulations followed. These changes made annual reporting of both quantitative and qualitative information relating to patient deaths a legal requirement in England.[17] The reporting mechanism was built into the NHS “Quality Accounts” system – where NHS secondary healthcare providers are legally required to produce a publicly available annual report about the quality of their services.[18] Guidance was not given on expected number of deaths, how to judge if a death was more likely than not due to problems in care, or on examples of learning, actions or how to assess impact of any actions. It was instead left to individual NHS trusts to decide how they would undertake and process these requirements. Guidance was given that NHS trust board leadership should ‘shares relevant learning across the organisation and with other services where the insight gained could be useful’.[15] and that trusts should ‘engage meaningfully with bereaved families and carers’.[19] It was not a statutory requirement to report on bereaved family and carer engagement or to report sharing of learning. Given the lack of consistency that led to the establishing the LfDs programme, this study sets out to analyse LfDs reporting, to ascertain if trusts are reporting as legally required, to evaluate the quality of reporting, and to understand if organisational learning in its truest sense is occurring.

For peer review only

METHODS

This is a qualitative and quantitative study of an NHS safety improvement programme. We undertook analysis of 2017/2018 quality account data from NHS secondary healthcare trusts in England. We excluded ambulance trusts (they are not required to report until 2020/2021). This study has been reported using Standards for Reporting Qualitative Research.[20]

Our objectives were to describe the quality of reporting, and to thematically analyse the reports to derive key learning for the NHS and beyond. We undertook analysis of LfDs as set out in the 2017 amendment to the NHS 2010 quality account regulations.

Our evaluation of the quality of reporting involved review of compliance of reports against regulation numbers 27.1 to 27.6 (table 1).[17] Where trusts did not fully report we sought to understand why this may have been the case from comments within the quality account itself. Data not found from the trust 2017/2018 quality account was not included in the analysis.

Table 1: NHS Quality Accounts LfDs Regulations [17]

Regulation number	Summary of regulatory requirement
**27.1**	The number of patients who have died during the annual reporting period
**27.2**	The number of the deaths (in 27.1) that have undergone a case record review or investigation
**27.3**	An estimate of the number of deaths in 27.2 which the provider judges to be more likely than not to have been due to problems in care, with explanation of method to assess this
**27.4**	What the provider has learnt from reviews/investigations in relation to deaths (in 27.3)
**27.5**	A description of the actions the provider has taken or will take in response to what they have learnt
**27.6**	An assessment of the impact of the actions (from 27.5)

In addition to statutorily required reporting we also looked for evidence within the 2017/18 LfDs report of family/carer engagement, which included evidence of involvement in learning and/or addressing family/carer concerns and/or appointing family liaison officer or similar as a result of a patient death. We also looked for evidence of sharing LfDs incidents both within the trust and more widely (for example with other organisations).

Quantitative analysis was undertaken and reported using descriptive statistics.

Qualitative evaluation to derive key learning themes was undertaken through document analysis using content and thematic analysis.[21] In order to collect data systematically, we first identified initial LfDs learning and action themes for reporting, and then developed a classification system for these. The first investigator (ZB) reviewed and analysed twenty 2017/2018 quality accounts, undertook open coding and combined this with information presented at the NHS Improvement London Network for Learning from Deaths event (October 2018), where themes (mixed learning and action) from London Trusts were

For peer review only

discussed. Following the initial review, we reviewed the further 202 NHS trust 2017/18 quality accounts. Each account was reviewed by the same reviewer twice to ensure full data capture. Researchers used the process of bracketing to reduce subjective analysis.[22] During data capture further themes emerged, were modified, merged and changed iteratively. Recurring themes were identified using a method of exploratory data analysis,[23] coding, identification of themes, recoding and using frequency charts. Data were captured in Microsoft excel.

Patient and public involvement

This study forms part of a larger programme of work which is overseen by a public and relatives steering group to improve relevance from the perspective of those affected by deaths in healthcare and to reduce biases from the healthcare staff researchers. The steering group have been involved in the planning, design and development of conclusions, through face-to-face meetings and email correspondence. The involvement of a steering group member in authoring this paper has significantly and positively influenced the reporting of this study, ensuring focus on reporting family involvement. The authors reflect that PPI has been essential to this study to ensure that the views of bereaved family members were central to the concerns examined. The reporting of patient and public involvement (PPI) has been undertaken using guidance for reporting involvement of patients and the public 2 – short form (GRIPP2-SF).[24]

For peer review only

RESULTS

Quality accounts were reviewed for all 222 secondary care trusts in England.

Quality of Reporting

98 out of 222 (44%) trusts reported all six statutory elements of the LfD reporting framework. Two trusts did not report any parts of the LfDs regulatory requirements.[25, 26] The total number of deaths reported (regulation 27.1) varied from 3 deaths to 7756 deaths .[27, 28] The number of case record reviews or investigations undertaken relative to the number of patient deaths in individual trusts varied between 0.2% and 100% of deaths; the average was 43.7%.

Number of deaths which the provider judges to be more likely than not to have been due to problems in care, with explanation of method used to assess this

There was variation between 0 and 13% in the number of deaths which the provider judged to be more likely than not to have been due to problems in care. 22 trusts did not report any figure in this section of the quality accounts, reasons given for this included:

- ‘data collection challenges’[29]
- ‘unable to provide a reliable figure’[30]
- ‘we do not carry out investigations with a view to determining whether the death was wholly or partly due to problems in the care provided’[31]
- ‘currently no research base on this for mental health services and no consistent accepted basis for calculating this data’[32]

111 out of 222 trusts (50%) noted the use of Structured Judgement Reviews (SJRs) (either Royal College of Physicians or Royal College of Psychiatrists) either alone or in combination with other forms of investigation or review to assess problems in care.[33] Trusts not using SJRs used a variety of other methods including: Confidential Enquiry into Stillbirths and Deaths in Infancy (CESDI) framework, Root Cause Analysis (RCA) and PReventable Incidents Survival and Mortality (PRISM) methodology.[34, 35]

Plans for assessment of impact

Regulation 26.6 asked trusts to undertake ‘an assessment of the impact of the actions’. 105 out of 222 trusts (47%) discussed assessment of impact. This includes trusts that had a plan of any sort including a future plan. Several trusts used audits and/or quality improvement projects to check that actions are implemented. One trust stated ‘Many of these actions are difficult to objectively assess in terms of their impact as they may relate to rare occurrences, which are difficult to meaningfully audit’.[36] The 47% of trusts who had a plan for assessment of impact does not include trusts that acknowledge the need to assess the impact but stated that it was too early to be able to undertake this (or words to this effect).[37, 38] Some trusts have reported the results of the assessment of impact that they have already undertaken.[39] Several trusts appear to have misunderstood, for example reiterating the purpose of the LfDs programme, instead of assessing impact.[40, 41]

For peer review only

Evidence involvement of family/carers in learning

In the 2017/18 LfDs reports 37 out of 222 trusts (17%) mentioned the involvement of families/carers either in the investigation process or in shared learning or that they communicate with/support/engage/consider families/carers after a patient dies.[42-44] A good example of working with families from one trust LfDs report states: 'The Trust continues to learn the importance of communication with families after a death has occurred and that through meaningful engagement after a death by inviting them to contribute to the terms of reference for investigations a more detailed, meaningful and richer account of the person's care and treatment is realised'.[45] One trust LfDs report discusses that as an action undertaken they sought to gain better education and training for staff about the importance of positive family engagement through expert external training.[46] 38 Trusts (17%) discussed as an 'action' that they plan to work with/communicate with/engage/support families/carers. Many of these trusts are the same trusts already undertaking family/carer engagement.

Evidence learning shared more widely

In the 2017/18 trust LfDs reports 106 out of 222 trusts (48%) have shared or plan to share the learning more widely within their own organisation, through a variety of communication mediums: Face to face meetings or events, trust intranet (as case studies, safety alerts, newsletters).[36, 44, 47] 17 out of 222 (8%) trusts have shared or plan to share the learning outside their organisation, with neighbouring trusts or other national organisations.[47-50]

Key Findings from the Reports

Lessons learnt

Regulation 27.4 asks trusts to describe 'what the provider has learnt from reviews/investigations in relation to deaths' where this was related to deaths which the provider judged to be more likely than not to have been due to problems in care (regulation 27.3). 25 out of 222 trusts (11%) did not report any lessons learnt from deaths; of these 25 trusts, 9 trusts had reported 1 or more death judged to be more likely than not due to problems in care, the other 16 trusts had either reported zero deaths judged to be more likely than not due to problems in healthcare or had not reported. However, 49 out of 222 trusts (22%) which reported that they had no deaths judged more likely than not due to problems in care, also reported lessons learnt, many caveating this with an explanation that they had learnt valuable lessons through the process of case note review/investigation. The most common learning themes from all trusts who reported learning can be found in table 2. An overview of the themes arising can be found in the frequency table (figure 1).

Table 2. The 5 most common learning themes across all trusts

Learning Themes	% of trusts citing theme
Poor communication (including language barrier and problems with handover)	46%
Problem in recognition and escalation of deteriorating patients	42%

End of life planning or treatment escalation planning not evident/incomplete	42%
Problems with documentation including consent, details patient team and NOK	41%
Lack of/problem with risk assessment/interventions	25%

Some trusts have undertaken analysis of their learning and described common themes.[51] Some have gone into great detail.[52] Others have described a specific case or cases.[53] Some trusts have identified learning and actions together, without differentiating the learning from the action. The lack of structure in reporting makes it difficult to always understand exactly what the problem was leading to the learning. This could reduce the transferability of the learning.[54] Some trusts identified 'Good practice' as learning points.[55] Occasionally trusts did not necessarily learn from patient deaths, but from the overall LfDs process.[56]

Actions taken or planned to be taken

Trusts were asked to undertake 'a description of the actions the provider has taken or will take in response to what they have learnt'. 30 out of the 222 trusts (14%) did not report any actions taken as a result of learning. One trust reported that they felt they were 'at too early a stage of development to be able to take actions from specific learning'.[37] The most common action themes from all trusts who reported actions can be found in table 3. An overview of the themes arising can be found in the frequency table (figure 2).

Table 3. The 5 most common action themes across all trusts

Action Themes	% of trusts citing theme
Review of process/Standard Operating Procedure (SOP)/pathway	58%
Highlight guidelines or protocols/policy use of guideline/policies or protocols/treatment bundle/toolkit	43%
Implementation programme of work/education/bundle	43%
Quality improvement work or similar	39%
Work to improve communication/collaboration/shared learning	28%

The level of detail with regards to actions taken varies greatly with some trusts listing some specific actions as bullet points.[39] Others trusts have described a specific case or cases.[57, 58]

For peer review only

DISCUSSION

This study demonstrates wide variation in both the quality of reporting and the findings from LfDs reports. Considering this is a new programme, introduced part-way through 2017/18, with limited guidance, the overall findings are somewhat encouraging. Nearly all trusts reported at least one or more element of the LfD reporting framework. Most trusts reported lessons learnt and/or actions taken, while less than half discussed assessment of impact. The lessons learnt were varied. The most common learning theme was poor communication, with the most common action being; review of process/Standard Operating Procedure (SOP)/pathway.

Quality of Reporting

Reporting variation may be due to differences in interpretation of the guidance and statutory requirements. There is no direct financial penalty for a trust not reporting some or all elements of the LfDs statutory requirements in their Quality Accounts. A penalty arises during CQC inspections, through assessment of implementation of LfDs.[59, 60]

The different approaches taken by trusts and the heterogeneity of data makes comparison difficult. The variation in the percentage of deaths being reviewed/investigated may be due to some trusts not having the capacity to review/investigate cases, collect and/or report accurately. Trusts with a very small number of deaths may find it easier to review all deaths than very large trusts. Some trusts have had mortality review processes in place for several years and have already been reviewing/investigating deaths, making implementation of the LfDs process easier since the structure for reviewing cases and personnel required are already in place. Some trusts may have felt at risk from negative attention by declaring total numbers of deaths and deaths judged more likely than not due to problems in care. Many trusts did however report despite the same risk. It is clear from the LfDs reports that several trusts, particularly some mental health and community trusts, did not feel that the guidance applied to them, however other similar trusts were able to comply with reporting. The results could suggest guidance was written with acute trusts in mind and perhaps need to be reconsidered for non-acute trusts. Similar findings were noted by the CQC in their report 'Learning from deaths: A review of the first year of NHS trusts implementing the national guidance'.[60]

The variation in deaths judged more likely than not due to problems in care is larger than those noted in previous studies.[2-6] It seems unlikely than many trusts would experience no deaths judged more likely than not due to problems in care. This could realistically be the case in specialist trusts where the absolute number of total deaths is very small, or community trusts with no inpatient beds, but seems unlikely in large acute trusts. Despite the improbability several acute trusts did report zero deaths judged more likely than not due to problems in care. Further work to understand why these trusts reported zero deaths should be undertaken.

The element of the statutory LfDs reporting that prompted poor responses from most trusts was 'An assessment of the impact of the actions' and describing how they would undertake this. The vast majority of trusts have answered this in a vague manner, seemingly through variable interpretation of the regulation. Improvements could be made by issuing further specific guidance in relation to this element of the reporting. Of the trusts who did manage to implement actions and assess impact this was often using quality improvement measurements. The use of quality improvement methodology is felt to be an important

For peer review only - http://bmjopen.bmj.com/site/about/guidelines.xhtml

overall indicator of quality by the CQC.[61] Guidance on evaluating the impact of interventions is widely available.[62, 63]

Collectively within the LfDs reports, there is much learning, some resulting in impactful actions and high-level organisational learning.[8] This learning could potentially be usefully shared across the NHS and internationally. Some NHS trusts appear to have disengaged with the programme. This study suggests a lack of shared learning from the LfDs reports particularly across organisations and a lack of family engagement, despite NHS guidance.[19] Since the involvement of families and sharing learning were not statutory requirements of LfDs reporting, they may be underrepresented in the LfDs reports, this should be investigated further. The apparent disparity in organisational learning and safety culture, results in inequity for families/carers. This should be addressed by the DHSC and associated national bodies. Since the oversight bodies which were established to support the programme in its initial stages have now been stood down this seems unlikely to happen.[64]

Key Findings from the Reports

Overall consistency with regards to identifying, reporting, investigating, learning from deaths in care and taking action has improved across most trusts. The continual process of learning, action and reflection which characterises effective organisational learning is essential to ensure the change necessary for safer healthcare. This can only be achieved where information and knowledge affecting patient safety is easily accessible to all members of healthcare staff, supporting an overall safety culture.[65, 66]

Only a small number of trusts did not report any learning, suggesting that most trusts were able to engage with this aspect of reporting. Many trusts have effectively described lessons learnt and actions taken. However, most of the LfDs report recommendations or actions are fairly non-specific; further detail of actions and their measurable impact would be helpful.

It is of concern that the majority of these lessons and recommended actions have previously been identified in national and international reports and inquiries, looking at the problems associated with preventable deaths. Similar problems found in this study are also highlighted in these reports; poor clinical monitoring, poor recognition of the deteriorating patient, diagnostic errors, poor communication, lack of end of life planning, lack of information sharing between services, inadequate drug and fluid management.[67-75] This suggests many of the same problems reoccur and that healthcare systems do not learn from previous failings and adds weight to the proposition that the NHS as a whole cannot become a learning organisation.[76] In view of this, it is reasonable to question whether the learning arising from LfDs reporting will result in meaningful change. If LfDs findings and recommendations are not implemented, systemic redundancy in the initiative is implied. While individual healthcare practitioners do need to take some responsibility, trusts and the DHSC should look at systems, such as institutional accountability and LfDs programme oversight to optimise outcomes and prevent errors from occurring. This lack of change adds to the growing body of evidence suggesting that traditional approaches to organisational learning in healthcare, such as learning from when things go wrong (safety-I) have limited effect and may suggest a role for increased learning from the patients who survive against the odds (safety-II).[77]

For peer review only

Recommendations

In view of the findings from this study, in order to improve reporting quality, our recommendations are as follows:

- A more structured LfDs reporting template, including all regulatory requirements should be implemented through the quality accounts
- NHSE/I specific guidance should be developed on how trusts can undertake 'an assessment of the impact of the actions'
- To reinstate LfDs robust regulatory reporting oversight in addition to CQC inspections

In order to improve 'learning and action' from deaths, our recommendations are:

- Annual collection and collation of all trust LfDs reporting for wider sharing
- Further investigation into how trusts currently involve bereaved families and carers
- Investment in leadership and support for NHS staff to enable a safety culture

For peer review only

CONCLUSION

This research shows that the LfDs programme has improved the way that NHS trusts identify, report, investigate and learn from deaths in care. However, more could be done to enhance and strengthen the programme impact, and to assess whether LfDs reporting reflects trust LfDs engagement.

On the basis of findings from the 2017/18 LfDs reports, national programmes led by multidisciplinary healthcare practitioners should be developed to tackle the most common problems which may have contributed to patient deaths. In the first instance programmes tackling the following issues should be developed or strengthened:

- Improving communication
- Involvement of families in care and in learning
- Processes to share learning (locally and nationally)

Further work is needed to understand which actions taken by trusts result in the biggest impact and for this learning to be shared. While LfDs can be difficult and emotive it is fundamental that healthcare systems ensure learning and impactful change occur.

ACKNOWLEDGMENTS

We would like to acknowledge the work of the Learning from Deaths: Learning and Action (LfDLaA) Public and Relatives steering group in this research. Patient and public involvement in this research was supported by the NIHR UCL Biomedical Research Centre.

For peer review only

REFERENCES

1 World Health Organisation 2019. Patient safety. Accessed July 2020. Available at: https://www.who.int/news-room/fact-sheets/detail/patient-safety
2 Levinson DR 2010. Adverse events in hospitals: national incidence among Medicare beneficiaries. Office of Inspector General for the Department of Health and Human Services. Accessed July 2020. Available at: https://oig.hhs.gov/oei/reports/oei-06-09-00090.pdf
3 Kobewka DM, Van Walraven C et al. The prevelance of potentially preventable deaths in an acute care hospital.: A retrospective cohort. Medicine. 2017 Feb;96(8):e6162. doi:1097/MD.0000000000006162
4 Hogan H, Healey F et al. Preventable deaths due to problems in care in English acute hospitals: retrospective case record review study. BMJ Quality & Safety 2012;21:737-745. doi:10.1136/bmjqs-2011-001159
5 Roberts AP, Morrow G et al. From research to practice: results of 7300 mortality retrospective case record reviews in four acute hospitals in the North-East of England. BMJ Open Qual.2017;6(2):e000123. doi:10.1136/bmjoq-2017-000123
6 Rogne T, Nordseth T et al. Rate of avoidable deaths in a Norwegian hospital trust as judged by retrospective chart review. BMJ Quality & safety. 2019 Jan;28(1):49-55. doi:10.1136/bmjqs-2018-008053
7 NHS Digital 2018. Summary Hospital-level Mortality Indicator (SHMI) - Deaths associated with hospitalisation, England, April 2017 - March 2018. Accessed July 2020. Available at: https://digital.nhs.uk/data-and-information/publications/clinical-indicators/shmi/archive/shmi-april-2017---march-2018
8 Fiol M, Lyles MA. Organizational Learning. The Academy of Management Review. Oct 1985;10(4):803-813
9 Salancik GR, Mendl JR. Corporate attributions as strategic illusions of management control. Administrative Science Quarterly 1984;29:238-254
10 Carroll JS, Edmondson AC. Leading organisational learning in health care. Qual Saf Health Care 2002;11:51–56. doi:10.1136/qhc.11.1.51
11 Mannion R, Braithwaite J. Where is Patient Safety Research and Practice Heading? A Response to Recent Commentaries. Int J Health Policy Manag 2019;8(2):136-137. doi: 10.15171/ijhpm.2018.112
12 Hamblin R, Shuker C Well spotted: but now you need to do something BMJ Quality & Safety Published Online First: 18 September 2020. doi: 10.1136/bmjqs-2019-010659
13 Mazars 2015. Independent review of deaths of people with a Learning Disability or Mental Health problem in contact with Southern Health NHS Foundation Trust April 2011 to March 2015. Accessed July 2020. Available at: https://www.england.nhs.uk/south/wp-content/uploads/sites/6/2015/12/mazars-rep.pdf
14 CQC 2016. Learning, candour and accountability. Accessed July 2020. Available at: https://www.cqc.org.uk/sites/default/files/20161213-learning-candour-accountability-full-report.pdf
15 NQB 2017. National Guidance on Learning from Deaths. Accessed July 2020. Available at: Accessed July 2020. Available at: https://www.england.nhs.uk/wp-content/uploads/2017/03/nqb-national-guidance-learning-from-deaths.pdf
16 NHS Improvement 2017. Implementing the Learning from Deaths framework: key requirements for trust boards. Accessed July 2020. Available at: https://improvement.nhs.uk/documents/1747/170921__Implementing_LfD_-_information_for_boards_JH_amend_3.pdf

17 UK Legislation 2017. The National Health Service (Quality Accounts) (Amendment) Regulations 2017. Accessed July 2020. Available at: http://www.legislation.gov.uk/uksi/2017/744/made
18 NHS 2019. About Quality Accounts. Accessed July 2020. Available at: https://www.nhs.uk/using-the-nhs/about-the-nhs/quality-accounts/about-quality-accounts/
19 NQB 2018. Learning from deaths: Guidance for NHS trusts on working with bereaved families and carers. Accessed Sept 2020. Available at: https://www.england.nhs.uk/wp-content/uploads/2018/08/learning-from-deaths-working-with-families-v2.pdf
20 O'Brien BC, Harris IB et al. Standards for reporting qualitative research: a synthesis of recommendations. Acad Med. 2014;89(9):1245-1251. doi:10.1097/ACM.0000000000000388
21 Bowen GA. 2009. Document Analysis as a Qualitative Research Method, Qualitative Research Journal. 9 (2). 27-40. DOI:10.3316/QRJ0902027
22 Tufford L, Newman P. Bracketing in Qualitative Research. Qualitative Social Work 2010;11:80-96. doi:10.1177/1473325010368316
23 Stebbins R. Exploratory Data Analysis. In: Given LM, The Sage Encyclopedia of Qualitative Research Methods, Vol. 1, Ed. 1. Sage 2008:325-326
24 Staniszewska S, Simera I et al. GRIPP2 reporting checklists: tools to improve reporting of patient and public involvement in research. BMJ 2017;358:j3453. doi: 10.1136/bmj.j3453
25 Worcestershire Acute Hospitals NHS Trust Quality Account 2017/18. Accessed July 2020. Available at: https://www.worcsacute.nhs.uk/stoma-care/2146-quality-account-2017-18/file
26 North Staffordshire Combined Healthcare NHS Trust Quality Account 2017/18. Accessed July 2020. Available at: http://389w1t43oria4ctlpf36b5lz-wpengine.netdna-ssl.com/wp-content/uploads/2018/10/Combined-Quality-Account-2017-18.pdf
27 Moorfields Eye Hospital NHS Foundation Trust Quality Account 2017/18. Accessed July 2020. Available at: https://www.moorfields.nhs.uk/sites/default/files/Moorfields%20annual%20report%202017-18.pdf
28 North East London Foundation Trust Quality Account 2017/18. Accessed July 2020. Available at: https://www.nelft.nhs.uk/download.cfm?doc=docm93jijm4n3126.pdf&ver=5090
29 North Middlesex University Hospital NHS Trust Quality Account 2017/18. Accessed July 2020. Available at: http://www.northmid.nhs.uk/Portals/0/201809%20-%20NMUH%20Final%20Quality%20Account%202017-18.pdf?ver=2018-09-18-125322-917
30 Royal Devon and Exeter NHS Foundation Trust Quality Account 2017/18. Accessed July 2020. Available at: https://www.rdehospital.nhs.uk/documents/trust/documents/annual-report-2017-2018.pdf
31 Black Country Partnership NHS Foundation Trust Quality Account 2017/18. Accessed July 2020. Available at: https://www.bcpft.nhs.uk/documents/about-us/key-documents/annual-report/1851-bcp-annual-report-2018/file
32 Sheffield Health and Social Care NHS Foundation Trust Quality Account 2017/18. Accessed July 2020. Available at: https://www.shsc.nhs.uk/sites/default/files/2019-12/Quality%20Report%202017-2018.pdf
33 Healthcare Quality Improvement Partnership 2018. Royal College of Physicians: National mortality case record review. Annual report. Accessed July 2020. Available at: https://www.rcplondon.ac.uk/projects/outputs/national-mortality-case-record-review-nmcrr-annual-report-2018
34 MBRRACE-UK 2017. Perinatal Confidential Enquiry. Confidential Enquiry into Stillbirths and Deaths in Infancy (CESDI) framework:19-20. Accessed July 2020. Available

For peer review only

at: https://www.npeu.ox.ac.uk/downloads/files/mbrrace-uk/reports/MBRRACE-UK%20Intrapartum%20Confidential%20Enquiry%20Report%202017%20-%20final%20version.pdf
35 Hogan H 2014. Preventable, Incidents, Survival and Mortality Study 2 (PRISM) Medical Record Review Manual. Accessed July 2020. Available at: https://improvement.nhs.uk/documents/1423/PRISM_2_Manual_V2_Jan_14.pdf
36 University Hospital Southampton NHS Foundation Trust Quality Account 2017/18. Accessed July 2020. Available at: https://www.uhs.nhs.uk/Media/SUHTInternet/AboutUs/AnnualReportsStrategiesandPlans/201718/Annual-report-2017-18.pdf
37 Royal United Hospitals Bath NHS Foundation Trust. Accessed July 2020. Available at: https://www.ruh.nhs.uk/about/annual_report/documents/Annual_Report_2017-18.pdf
38 Great Western Hospitals NHS Foundation Trust Quality Account 2017/18. Accessed July 2020. Available at: https://www.gwh.nhs.uk/media/309191/gwhnhsftannualqualityaccounts2017-18.pdf
39 Salisbury NHS Foundation Trust Quality Account 2017/18. Accessed July 2020. Available at: https://www.salisbury.nhs.uk/AboutUs/TrustReportsAndReviews/Documents/SalisburyNHSFoundationTrustQualityAccount2017_2018.pdf
40 Gloucestershire Hospitals NHS Foundation Trust Quality Account 2017/18. Accessed July 2020. Available at: https://www.gloshospitals.nhs.uk/media/documents/QA-A4-17-18.pdf
41 Isle of Wight NHS Trust Quality Account 2017/18. Accessed July 2020. Available at: https://www.iow.nhs.uk/Downloads/Quality%20Account/Quality%20Accout%202017-18.pdf
42 Tameside and Glossop Integrated Care NHS Foundation Trust Quality Account 2017/18. Accessed July 2020. Available at: https://www.tamesidehospital.nhs.uk/Documents/AnnualReport20172018.pdf
43 Buckinghamshire Healthcare NHS Trust Quality Account 2017/18. Accessed July 2020. Available at: https://www.buckshealthcare.nhs.uk/Downloads/quality-account/08%20Quality%20Accounts_2017_18.pdf
44 Birmingham Women's and Children's NHS Foundation Trust Quality Account 2017/18. Accessed July 2020. Available at: https://bwc.nhs.uk/download.cfm?doc=docm93jijm4n2490.pdf&ver=3456
45 Pennine Care NHS Foundation Trust Quality Account 2017/18. Accessed July 2020. Available at: https://www.penninecare.nhs.uk/application/files/4615/6172/0636/Quality_account_2017-18.pdf
46 South Staffordshire and Shropshire Healthcare NHS Foundation Trust Quality Account 2017/18. Accessed July 2020. Available at: https://improvement.nhs.uk/documents/4294/South_Staffordshire_Shropshire_Healthcare_Annual_Report_and_Accounts_2017-18.PDF
47 Royal Free London NHS Foundation Trust Quality Account 2017/18. Accessed July 2020. Available at: https://www.royalfree.nhs.uk/uploads/RF_quality_report_2017-18.pdf
48 South west Yorkshire Partnership NHS Foundation Trust Quality Account 2017/18. Accessed July 2020. Available at: https://www.southwestyorkshire.nhs.uk/wp-content/uploads/2018/08/SWYPT-annual-report-and-accounts-2017-18-FINAL.pdf
49 Northumbria Healthcare NHS Foundation Trust Quality Account 2017/18. Accessed July 2020. Available at: https://www.northumbria.nhs.uk/wp-content/uploads/2018/07/FINAL-NHFT-ARA-2017-18.pdf

For peer review only

50 Greater Manchester Mental Health NHS Foundation Trust Quality Account 2017/18. Accessed July 2020. Available at: https://www.gmmh.nhs.uk/download.cfm?doc=docm93jijm4n3649.pdf&ver=7138
51 The Christie NHS Foundation Trust Quality Account 2017/18. Accessed July 2020. Available at: https://www.christie.nhs.uk/media/5682/the-christie-nhs-ft-quality-report-2017-18.pdf
52 Northumberland, Tyne & Wear NHS Foundation Trust Quality Account 2017/18. Accessed July 2020. Available at: https://improvement.nhs.uk/documents/4419/Northumberland_Tyne___Wear_NHS_FT_Annual_Report_and_Accounts_2017-18.PDF
53 The Walton Centre NHS Foundation Trust Quality Account 2017/18. Accessed July 2020. Available at: https://www.thewaltoncentre.nhs.uk/uploadedfiles/THE%20WALTON%20CENTRE%20FINAL%20MERGED%20ANNUAL%20REPORT%20ACCOUNTS%20QUALITY%20ACCOUNT%202017_18.pdf
54 Kent & Medway NHS and Social Care Partnership Trust Quality Account 2017/18. Accessed July 2020. Available at: https://www.kmpt.nhs.uk/media/1234/quality-account-17-18.pdf
55 Bradford Teaching Hospitals NHS Foundation Trust Quality Account 2017/18. Accessed July 2020. Available at: https://www.bradfordhospitals.nhs.uk/wp-content/uploads/2018/07/Annual-Report-and-Accounts-2017-18.pdf
56 Maidstone and Tunbridge Wells NHS Trust Quality Accounts 2017/18. Accessed July 2020. Available at: https://www.mtw.nhs.uk/wp-content/uploads/2018/06/MTW-Quality-Accounts-2018.pdf
57 Cambridge University Hospitals NHS Foundation Trust Quality Account 2017/18. Accessed July 2020. Available at: https://www.cuh.nhs.uk/sites/default/files/Annual%20Report%20and%20Accounts%20201718.pdf
58 Camden and Islington NHS Foundation Trust Quality Account 2017/18. Accessed July 2020. Available at: https://www.cuh.nhs.uk/sites/default/files/Annual%20Report%20and%20Accounts%20201718.pdf
59 CQC 2018. Inspection framework: NHS trusts and foundation trusts. Trust-wide well-led. Accessed July 2020. Available at: https://www.cqc.org.uk/sites/default/files/20200115_Trust_wide_well_led_inspection_framework_V7.pdf
60 CQC 2019. Learning from deaths: A review of the first year of NHS trusts implementing the national guidance Accessed July 2020. Available at: https://www.cqc.org.uk/sites/default/files/20190315-LfD-Driving-Improvement-report-FINAL.pdf
61 CQC 2018. Quality improvement in hospital trusts. Accessed July 2020. Available at: https://www.cqc.org.uk/sites/default/files/20180911_QI_hospitals_FINAL.pdf
62 NHS 2015. How to understand and measure impact. Accessed Sept 2020. Available at: https://www.england.nhs.uk/wp-content/uploads/2015/06/bcf-user-guide-04.pdf.pdf
63 Clarke Geraldine M, Conti Stefano et al. Evaluating the impact of healthcare interventions using routine data BMJ 2019;365:l2239. doi: 10.1136/bmj.I2239
64 RCP 2019. National Mortality Case Record Review Programme. Accessed July 2020. Available at: https://www.rcplondon.ac.uk/projects/national-mortality-case-record-review-programme

For peer review only

65 Carroll JS, Edmondson AC. Leading organisational learning in healthcare. BMJ Quality & Safety 2002;11(1):51-6. doi: 10.1136/qhc.11.1.51
66 Mannion R, Konteh FH, Davies HTO. Assessing organisational culture for quality and safety improvement: a national survey of tools and tool use. BMJ Quality & Safety 2009;153:153-6. doi: 10.1136/qshc.2007.024075
67 National Confidential Enquiry into Patient Outcomes and Deaths (NCEPOD) 2018. Themes and recommendations common to all hospital specialties. Accessed July 2020. Available at: https://www.ncepod.org.uk/CommonThemes.pdf
68 NHS England 2014. Patient safety alert. Stage three: directive. Improving medication error incident reporting and learning. Accessed July 2020. Available at: https://www.england.nhs.uk/wp-content/uploads/2014/03/psa-sup-info-med-error.pdf
69 Singh H, Schiff GD, Graber ML, Onakpoya I, Thompson MJ. The global burden of diagnostic errors in primary care. BMJ Qual Saf 2017;26:484-494. doi: 10.1136/bmjqs-2016-005401
70 Oates A. Learning from suicide-related claims. A thematic review of NHS Resolution data. NHS Resolution. September 2018. Accessed July 2020. Available at: https://resolution.nhs.uk/wp-content/uploads/2018/09/NHS-Resolution_learing_from_suicide_claims_148pp_ONLINE1.pdf
71 Government of Western Australia, Department of Health 2019. From death we learn 2018. Accessed July 2020. Available at: https://ww2.health.wa.gov.au/~/media/Files/Corporate/Reports%20and%20publications/PDF/From-Death-We-Learn-2018.pdf
72 PHSO 2014. An avoidable death of a three-year-old child from sepsis. Accessed July 2020. Available at: https://www.ombudsman.org.uk/sites/default/files/An_avoidable_death_of_a_three_year_old_child_from_sepsis%20_report.pdf
73 Francis R. Report of the Mid Staffordshire NHS Foundation Trust Public Inquiry: Executive summary. Department of Health, 2013. Accessed July 2020. Available at: https://assets.publishing.service.gov.uk/government/uploads/system/uploads/attachment_data/file/279124/0947.pdf
74 Kirkup B. The Report of the Morecambe Bay Investigation. Department of Health, 2015. Accessed July 2020. Available at: https://assets.publishing.service.gov.uk/government/uploads/system/uploads/attachment_data/file/408480/47487_MBI_Accessible_v0.1.pdf
75 Walshe K, Higgins J. The use and impact of inquiries in the NHS. BMJ 2002;325(7369): 895–900. doi: 10.1136/bmj.325.7369.895
76 Sheaff R, Pilgrim D. Can learning organisations survive in the newer NHS? Implementation science 2006:1(27). doi: 10.1186/1748-5908-1-27
77 Sujan M. A Safety-II perspective on organisational learning in healthcare organisations. Int J Health Policy Manag 2018;7(7):662-666. doi: 10.15171/ijhpm.2018.16

For peer review only

Footnotes

Contributors

ZB conceived and designed the study, undertook analysis, interpretation of the data and drafted the manuscript.
CVP provided critical input to the design, analysis, interpretation of the data and revised the manuscript critically.
DB provided critical input to the design, analysis, interpretation of the data and revised the manuscript critically.
SRM provided critical input to the design, analysis, interpretation of the data and revised the manuscript critically.
The corresponding author attests that all listed authors meet authorship criteria and that no others meeting the criteria have been omitted. ZB is the guarantor.

Funding

This study was supported by the National Institute of Health Research (NIHR) University College London (UCL) Biomedical Research Centre Patient and Public Involvement starter funding (BRC617/PPI/ZB/104990).

Competing interests

ZB worked at NHS Improvement in the medical directorate from August 2017 to August 2018, during which time she undertook some work on the LfDs programme. SRM is the National Clinical Director for Critical and Perioperative Care for NHS England/NHS Improvement, SRM has no link with the LfDs programme. CVP and DB do not have any competing interests to declare. All authors have completed the ICMJE uniform disclosure form at www.icmje.org/coi_disclosure.pdf

Ethical approval

This study is an analysis of publicly available data and does not require ethical approval.

Data availability statement

All the data for this study is publicly available from the 2017/18 NHS Quality Accounts.

For peer review only

Figure Legends

Figure 1. Frequency table of lessons learnt (all trusts; n=222)

A – Problem in recognition & escalation of deteriorating patients
B – Lack of or awareness of or following protocol/guideline/bundle
C – Problem in assessment or experience related to learning disabilities
D – Poor communication (including language barrier & problems with handover)
E – Problem with end-of-life planning or treatment escalation planning
F – Problem with death certification or confirming death
G – Problem with discharge (timing/letters/delay/information for patients)
H – Difficulty accessing support services/ Out of Hours services/ Specialist services
I – Problem/lack of risk assessment/interventions
J – Lack of knowledge of hospital layout/equipment
K – Problem with transfers
L – Problem assessing/providing nutrition/fluids/electrolytes
M – Lack of senior/consultant review, input, planning
N – Excellent/good care/management
O – Prompt senior review
P – Good communication/collaboration/teamwork
Q – Lack of clinical knowledge, consideration differential diagnosis or seeking advise
R – Problem with/lack of prescribing or side-effects or administration of medications
S – Problem with 'Duty of Candour'
T – Delay to acting on results
U – Problems with documentation including consent
V – Delay/problem in requesting or interpretation of investigations
W – Lack of/problem with monitoring/observations/recording
X - Lack of/or problem with sharing information with other providers/services/specialties
Y – Delay in reviewing patient
Z – Delay in treatment/incomplete management including care plans and pain management
AA – Poor continuity of care/team work
AB – Concerns with pre-hospital care (for example in residential settings or wider societal issues)
AC – Lack of familiarity with or standardisation or availability of equipment
AD – Problem related to workforce or staffing or supervision of staff
AE – Misfiled documents/lost notes/problems in storage or access of notes/scans
AF – Problem with recognition/management of Acute Kidney Injury
AG – Lack of Multidisciplinary Team involvement/discussion/decision
AH – Problem with competency or complication in undertaking procedure/operation
AI – Problem related to infection control
AJ – Lack of/problem with assessment of mental health needs and/or follow-up
AK – Problem related to appropriateness of patient ward allocation or relocation
AL – Problem with pre-op assessment/peri-op management
AM – Problem with capacity/flow/hospital of department pressures (including A&E)
AN – Deviation from treatment plan or plan not linked with clinical record
AO - Follow-up planning not evident or incomplete/problem with follow-up
AP – Problem related to the management of physical health problem in the mental health setting
AQ – Problem due to patient not wanting to/unable to engage with treatment (with capacity)
AR – Problem after death (related to post-mortem/forensic services or investigation)
AS – Problem with the recognition/management of drug/alcohol withdrawal/recovery
AT – Lack of supervision or safe accommodation for vulnerable patient
AU – Lack of/problem with engagement with/support of families/carers

For peer review only - http://bmjopen.bmj.com/site/about/guidelines.xhtml

Figure 2. Frequency table of actions taken (all trusts; n=222)

A – Work to improve communication/collaboration/shared learning
B – Improved end-of-life planning (including communication)
C – Improved effectiveness of handover
D– Highlight or new or use of guidelines/protocols/policy/protocol/treatment bundle/toolkits
E – Improved mortality review process
F – Undertake or improve risk assessment/governance process/reporting system
G – Review of process/SOP/pathway/audit process
H – Quality improvement work or similar
I – 'Raising awareness' or 'Importance of' or 'reflecting on' (not qualified)
J – Implementation of a programme of work or education (including simulation & induction)
K – Raising awareness (with specific example – 'nursing dashboard', 'case presentation')
L – Use of technology (for example electronic recording of observations)
M – Rota adjusted to provide better cover or extra lists/sessions
N – Working/communicating with/supporting families (not end-of-life planning)
O – 'More effective', 'continued efforts', 'seeking advice' 'requirement to review/introduce' (not qualified)
P – Solution involving medical examiner role
Q – Improved senior/consultant involvement (with specific examples)
R – External or internal (peer review) mortality/governance review or investigation
S – Identification of high-risk patients early
T – Extend post-op recovery monitoring
U – Improved documentation/coding
V – Follow-up of action plans
W – Plan to improve sharing of learning
X – Ensure Early Warning System in place/utilised correctly
Y – Improvement of results reporting & acknowledgement process/archiving results/scans
Z – Multidisciplinary team/programme of work set-up to address specific problem
AA – Seek-out and follow expert advise
AB – Improve review methodology (Structured Judgement Review training or case selection as per Royal College of Physicians guidance)
AC – Develop regional Learning from Deaths network or similar
AD – Negotiate with coroner for earlier post-mortem reports
AE – Increase emergency operating capability (such as additional emergency theatre availability)
AF – Improvement to bereavement facilities
AG – Improved infection control measures (such as isolation ward for suspected transmissible infectious diseases)
AH – Supervision discussions/support/feedback for those involved in incidents
AI – Improved cross-specialty collaboration
AJ – Increased specialist equipment availability or specialist teams or specialist roles
AK – Increased engagement in LeDeR process

For peer review only

Figure 1. Frequency table of lessons learnt (all trusts; n=222)

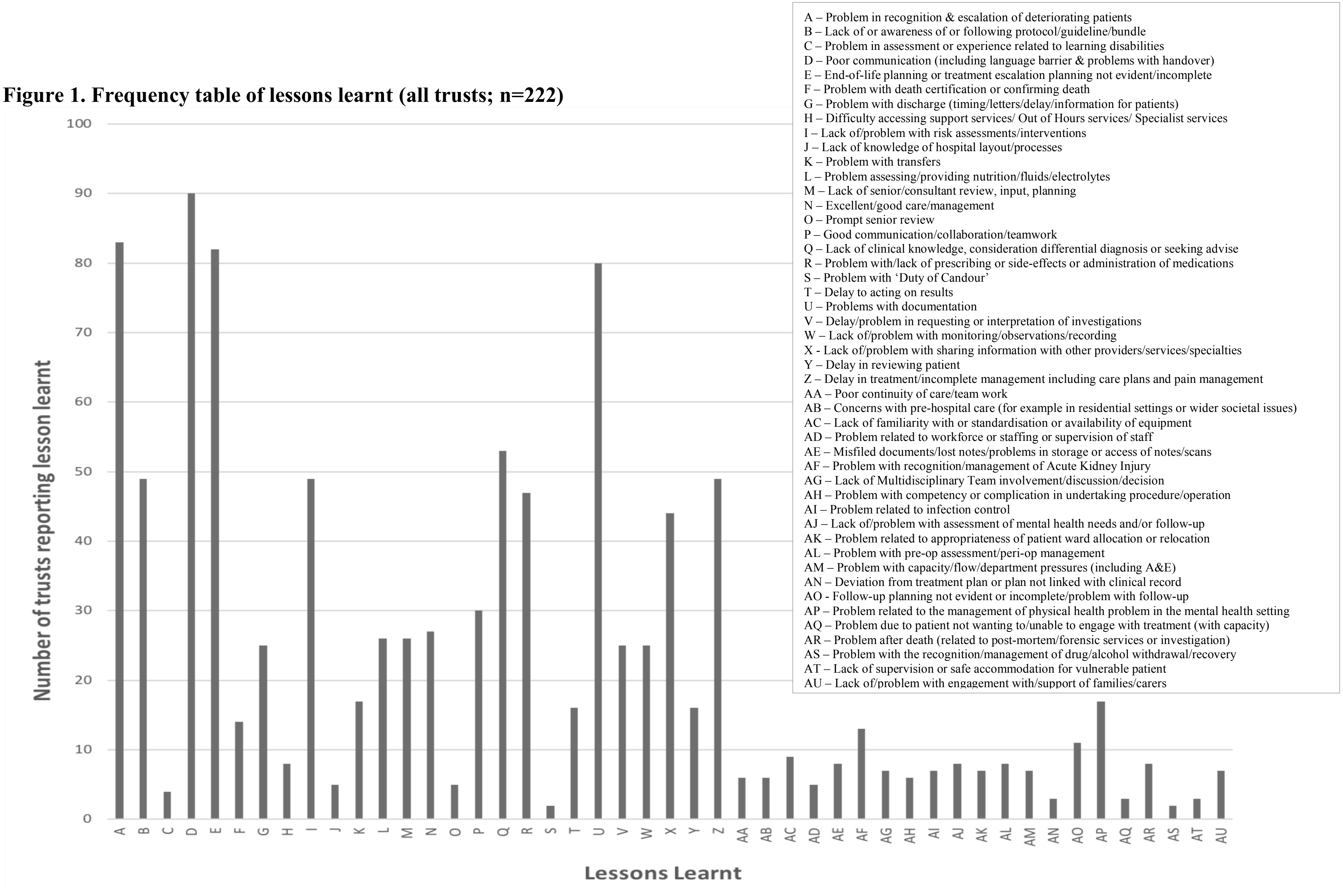

A – Work to improve communication/collaboration/shared learning
B – Improved end-of-life planning (including communication)
C – Improved effectiveness of handover
D– Highlight or produce guidelines/protocols/policies/treatment bundle/toolkits
E – Improved mortality review process
F – Undertake or improve risk assessment/governance process/reporting system
G – Review of process/SOP/pathway
H – Quality improvement work or similar
I – 'Raising awareness' or 'Importance of' or 'reflecting on' (not qualified)
J – Implementation programme of training/ education
K – Raising awareness (with specific example – 'nursing dashboard', 'case presentation')
L – Use of technology (for example electronic recording of observations)
M – Rota adjusted to provide better cover or extra lists/sessions
N – Working/communicating with/supporting families (not end-of-life planning)
O – 'More effective', 'continued efforts', 'seeking advice' 'requirement to review/introduce' (not qualified)
P – Solution involving medical examiner role
Q – Improved senior/consultant involvement (with specific examples)
R – External or internal (peer review) mortality/governance review or investigation
S – Identification of high-risk patients early
T – Extend post-op recovery monitoring
U – Improved documentation/coding
V – Follow-up of action plans
W – Plan to improve sharing of learning
X – Ensure Early Warning System in place/utilised correctly
Y – Improvement of results reporting & acknowledgement process/archiving results/scans
Z – Multidisciplinary team/programme of work set-up to address specific problem
AA – Seek-out and follow expert advise
AB – Improve review methodology (Structured Judgement Review training or case selection
AC – Develop regional Learning from Deaths network or similar
AD – Negotiate with coroner for earlier post-mortem reports
AE – Increase emergency operating capability
AF – Improvement to bereavement facilities
AG – Improved infection control measures (such as isolation ward)
AH – Supervision discussions/support/feedback for those involved in incidents
AI – Improved cross-specialty collaboration
AJ – Increased specialist equipment availability or specialist teams or specialist roles
AK – Increased engagement in LeDeR process

Figure 2. Frequency table of actions taken (all trusts; n=222)

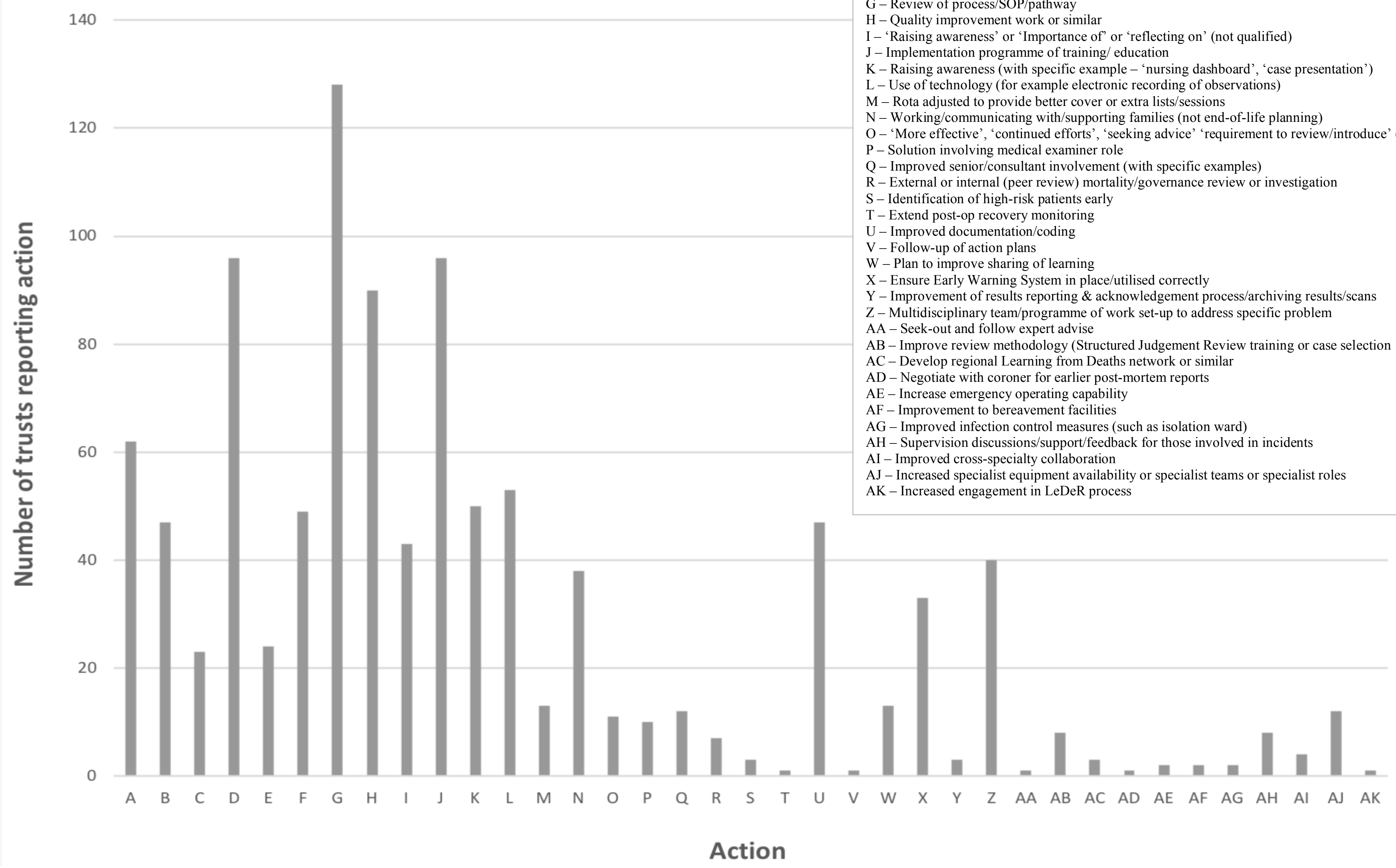

No.	Topic	Item
	**Title and abstract**	
S1	Title	Concise description of the nature and topic of the study Identifying the study as qualitative or indicating the approach (e.g., ethnography, grounded theory) or data collection methods (e.g., interview, focus group) is recommended
S2	Abstract	Summary of key elements of the study using the abstract format of the intended publication; typically includes background, purpose, methods, results, and conclusions
	**Introduction**	
S3	Problem formulation	Description and significance of the problem/phenomenon studied; review of relevant theory and empirical work; problem statement
S4	Purpose or research question	Purpose of the study and specific objectives or questions
	**Methods**	
S5	Qualitative approach and research paradigm	Qualitative approach (e.g., ethnography, grounded theory, case study, phenomenology, narrative research) and guiding theory if appropriate; identifying the research paradigm (e.g., postpositivist, constructivist/ interpretivist) is also recommended; rationale[b]
S6	Researcher characteristics and reflexivity	Researchers' characteristics that may influence the research, including personal attributes, qualifications/experience, relationship with participants, assumptions, and/or presuppositions; potential or actual interaction between researchers' characteristics and the research questions, approach, methods, results, and/or transferability
S7	Context	Setting/site and salient contextual factors; rationale[b]
S8	Sampling strategy	How and why research participants, documents, or events were selected; criteria for deciding when no further sampling was necessary (e.g., sampling saturation); rationale[b]
S9	Ethical issues pertaining to human subjects	Documentation of approval by an appropriate ethics review board and participant consent, or explanation for lack thereof; other confidentiality and data security issues
S10	Data collection methods	Types of data collected; details of data collection procedures including (as appropriate) start and stop dates of data collection and analysis, iterative process, triangulation of sources/methods, and modification of procedures in response to evolving study findings; rationale[b]
S11	Data collection instruments and technologies	Description of instruments (e.g., interview guides, questionnaires) and devices (e.g., audio recorders) used for data collection; if/how the instrument(s) changed over the course of the study
S12	Units of study	Number and relevant characteristics of participants, documents, or events included in the study; level of participation (could be reported in results)
S13	Data processing	Methods for processing data prior to and during analysis, including transcription, data entry, data management and security, verification of data integrity, data coding, and anonymization/deidentification of excerpts
S14	Data analysis	Process by which inferences, themes, etc., were identified and developed, including the researchers involved in data analysis; usually references a specific paradigm or approach; rationale[b]
S15	Techniques to enhance trustworthiness	Techniques to enhance trustworthiness and credibility of data analysis (e.g., member checking, audit trail, triangulation); rationale[b]
	**Results/findings**	
S16	Synthesis and interpretation	Main findings (e.g., interpretations, inferences, and themes); might include development of a theory or model, or integration with prior research or theory
S17	Links to empirical data	Evidence (e.g., quotes, field notes, text excerpts, photographs) to substantiate analytic findings
	**Discussion**	
S18	Integration with prior work, implications, transferability, and contribution(s) to the field	Short summary of main findings; explanation of how findings and conclusions connect to, support, elaborate on, or challenge conclusions of earlier scholarship; discussion of scope of application/ generalizability; identification of unique contribution(s) to scholarship in a discipline or field
S19	Limitations	Trustworthiness and limitations of findings
	**Other**	
S20	Conflicts of interest	Potential sources of influence or perceived influence on study conduct and conclusions; how these were managed
S21	Funding	Sources of funding and other support; role of funders in data collection, interpretation, and reporting

[a]The authors created the SRQR by searching the literature to identify guidelines, reporting standards, and critical appraisal criteria for qualitative research; reviewing the reference lists of retrieved sources; and contacting experts to gain feedback. The SRQR aims to improve the transparency of all aspects of qualitative research by providing clear standards for reporting qualitative research.
[b]The rationale should briefly discuss the justification for choosing that theory, approach, method, or technique rather than other options available, the assumptions and limitations implicit in those choices, and how those choices influence study conclusions and transferability. As appropriate, the rationale for several items might be discussed together.

ACADEMIC MEDICINE

Standards for Reporting Qualitative Research: A Synthesis of Recommendations

O'Brien, Bridget C.; Harris, Ilene B.; Beckman, Thomas J.; Reed, Darcy A.; Cook, David A.
Academic Medicine89(9):1245-1251, September 2014.
doi: 10.1097/ACM.0000000000000388

Standards for Reporting Qualitative Research (SRQR)a

Wolters Kluwer
Copyright © 2020 by the Association of American Medical Colleges

BMJ Open

NHS 'Learning from Deaths' reports: A qualitative and quantitative document analysis of the first year of a countrywide patient safety programme

Journal:	*BMJ Open*
Manuscript ID	bmjopen-2020-046619.R1
Article Type:	Original research
Date Submitted by the Author:	24-Mar-2021
Complete List of Authors:	Brummell, Zoe; University College London Hospitals NHS Foundation Trust, Anaesthetics Department<br>Vindrola-Padros, Cecilia; University College London, Department of Targeted Intervention; UCL, UCL<br>Braun, Dorit; Advisor/Lived experience<br>Moonesinghe, S. Ramani; UCL/UCLH Surgical Outcomes Research Centre, Centre for Perioperative Medicine, Department for Targeted Intervention, Division of Surgery and Interventional Science; Health Services Research Centre, , National Institute for Academic Anaesthesia, Royal College of Anaesthetists
**Primary Subject Heading**:	Health policy
Secondary Subject Heading:	Health services research, Medical management, Qualitative research
Keywords:	Health & safety < HEALTH SERVICES ADMINISTRATION & MANAGEMENT, Health policy < HEALTH SERVICES ADMINISTRATION & MANAGEMENT, Organisational development < HEALTH SERVICES ADMINISTRATION & MANAGEMENT, Quality in health care < HEALTH SERVICES ADMINISTRATION & MANAGEMENT, QUALITATIVE RESEARCH

SCHOLARONE™
Manuscripts

I, the Submitting Author has the right to grant and does grant on behalf of all authors of the Work (as defined in the below author licence), an exclusive licence and/or a non-exclusive licence for contributions from authors who are: i) UK Crown employees; ii) where BMJ has agreed a CC-BY licence shall apply, and/or iii) in accordance with the terms applicable for US Federal Government officers or employees acting as part of their official duties; on a worldwide, perpetual, irrevocable, royalty-free basis to BMJ Publishing Group Ltd ("BMJ") its licensees and where the relevant Journal is co-owned by BMJ to the co-owners of the Journal, to publish the Work in this journal and any other BMJ products and to exploit all rights, as set out in our licence.

The Submitting Author accepts and understands that any supply made under these terms is made by BMJ to the Submitting Author unless you are acting as an employee on behalf of your employer or a postgraduate student of an affiliated institution which is paying any applicable article publishing charge ("APC") for Open Access articles. Where the Submitting Author wishes to make the Work available on an Open Access basis (and intends to pay the relevant APC), the terms of reuse of such Open Access shall be governed by a Creative Commons licence – details of these licences and which Creative Commons licence will apply to this Work are set out in our licence referred to above.

Other than as permitted in any relevant BMJ Author's Self Archiving Policies, I confirm this Work has not been accepted for publication elsewhere, is not being considered for publication elsewhere and does not duplicate material already published. I confirm all authors consent to publication of this Work and authorise the granting of this licence.

For peer review only

NHS 'Learning from Deaths' reports: A qualitative and quantitative document analysis of the first year of a countrywide patient safety programme

Authors:

Zoe Brummell (0000-0002-6296-5049), Researcher/Clinician, Department of Anaesthesia, University College London Hospitals NHS Foundation Trust, London, NW1 2BU

Cecilia Vindrola-Padros (0000-0001-7859-1646), Senior Researcher, Department of Targeted Intervention, University College London, Charles Bell House, 43-45 Foley Street, London, W1W 7TS

Dorit Braun, Advisor/Lived experience, Learning from Deaths: Learning and Action Public and Relatives Steering Group member

S. Ramani Moonesinghe (0000-0002-6730-5824), Professor, Centre for Perioperative Medicine, Department for Targeted Intervention, University College London, Charles Bell House, 43-45 Foley Street, London, W1W 7TS

Corresponding author:

Zoe Brummell (0000-0002-6296-5049), Researcher/Clinician, Department of Anaesthesia, University College London Hospitals NHS Foundation Trust, London, England, NW1 2BU

z.brummell@nhs.net

For peer review only

ABSTRACT

Objectives

To review how NHS Secondary Care Trusts (NSCTs) are using the Learning from Deaths (LfDs) programme to learn from and prevent, potentially preventable deaths.

Introduction

Potentially preventable deaths occur worldwide within healthcare organisations. In England, inconsistencies in how NSCTs reviewed, investigated and shared learning from deaths, resulted in the introduction of national guidance on 'Learning from Deaths' in 2017. This guidance provides a 'framework for identifying, reporting, investigating and learning from deaths'. Amendments to NHS Quality Account regulations, legally require NSCTs in England to report quantitative and qualitative information relating to patient deaths annually. The programme intended NSCTs would share this learning and take measurable action to prevent future deaths.

Method

We undertook qualitative and quantitative secondary data, document analysis of all NSCTs LfDs reports within their 2017/18 Quality Accounts (n=222).

Results

All statutory elements of LfDs reporting were reported by 98 out of 222 (44%) NSCTs. The percentage of deaths judged more likely than not due to problems in healthcare was between 0% and 13%. The majority of NSCTs (89%) reported lessons learnt; the most common learning theme was poor communication. 106 out of 222 NSCTs (48%) have shared or plan to share the learning within their own organisation. The majority of NSCTs (86%) reported actions taken and 47% discussed or had a plan for assessment of impact. 37 out of 222 NSCTs (17%) mentioned involvement of bereaved families.

Conclusions

The wide variation in reporting demonstrates that some NSCTs have engaged fully with LfDs, while other NSCTs appear to have disengaged with the programme. This may reveal a disparity in organisational learning and patient safety culture which could result in inequity for bereaved families. Many themes identified from the LfDs reports have previously been identified by national and international reports and inquiries.

For peer review only

STRENGTHS AND LIMITATIONS OF THIS STUDY

This is the first study to our knowledge to analyse Learning from Deaths (LfDs) reporting.

Quality Accounts from all NSCTs in England legally required to report LfDs were included in both the quantitative and qualitative analysis. Including all NSCTs in the qualitative analysis has ensured complete and thorough data capture. Despite attempts to minimise inherent researcher bias, the qualitative analysis may have been influenced by to some extent.

This study has ensured the inclusion of views from bereaved relatives, through Patient and Public Involvement. The authors conclude that these views are essential to improving patient safety.

This is an analysis of the very first year of LfDs reporting and reports could underrepresent current NSCT engagement in the LfDs process.

NSCTs may be undertaking elements of the LfDs programme that were not statutory reporting requirements such as family/carer engagement, but not reporting on these as it was not a regulatory requirement.

For peer review only

INTRODUCTION

Globally, adverse events while receiving healthcare is a leading cause of morbidity and mortality.[1] The percentage of preventable or potentially preventable deaths is likely to lie somewhere between 0.5% and 8.4% of hospital deaths.[2-6] In England between April 2017 and end of March 2018 there were 299,000 deaths occurring in hospital or within 30 days of discharge, this amounts to an estimate of between 1,495 and 25,116 potentially preventable deaths.[7] There is a moral imperative for healthcare organisations to learn from these deaths and take measurable action to prevent potentially preventable deaths.

Healthcare organisations are made up of individuals who have the ability to learn: however organisational learning is 'more than the sum of individual learning' and is distinct from unreflective action taking.[8] It is more than simply creating change for change's sake, as an 'illusion of learning'.[9] Organisational learning is the ability to apply knowledge and understanding to increase effective organisational action.[8, 10] Effective organisational learning is crucial to improve patient safety and probably requires both safety-I (understanding why things go wrong) and safety-II (understanding why things go right) approaches.[11, 12] Central regulation and performance management may have some effect on improving care, but quality improvement, leadership, public engagement, proper resourcing, education, and training are needed for a safer health service.[12]

In April 2016 an independent review demonstrated a lack of systematic approach and meaningful change in response to unexpected deaths at Southern Health NHS Foundation Trust.[13] The Care Quality Commission (CQC), which is responsible for monitoring, inspection and regulation of healthcare services within England, conducted a wider review into the investigations of deaths. They found inconsistencies in the way NHS Secondary Care Trusts (NSCTs) became aware of, investigated and shared learning from deaths.[14] In response, the NHS launched a new programme of work to improve standards. This included national guidance on 'Learning from Deaths' (LfDs), providing a framework for NSCTs on 'identifying, reporting, investigating and learning from deaths in care'. The objectives of the guidance included supporting the NHS in England to develop an understanding of why deaths arising from problems in care occur, with the aim of ensuring that findings are shared and acted upon, to prevent recurrence.[15] In July 2017 guidance was published on implementing the LfDs framework at NSCT board level,[16] and amendments to statutory regulations followed. These changes made annual reporting of both quantitative and qualitative information relating to patient deaths a legal requirement in England.[17] The reporting mechanism was built into the NHS "Quality Accounts" system – where NSCTs are legally required to produce a publicly available annual report about the quality of their services (United Kingdom government legislation).[18]

Guidance was not given on expected number of deaths, how to judge if a death was more likely than not due to problems in care, or on examples of learning, actions or how to assess impact of any actions. It was instead left to individual NSCTs to decide how they would undertake these requirements. Guidance was given that NSCT board leadership should 'shares relevant learning across the organisation and with other services',[15] and that NSCTs should 'engage meaningfully with bereaved families and carers'.[19] It was not a statutory requirement to report on bereaved family and carer engagement or to report sharing of learning. This study analyses if NSCTs are reporting as legally required, evaluates the quality of reporting, and determines whether there is evidence of effective organisational learning, sharing of learning and engagement with bereaved families and carers.

For peer review only - http://bmjopen.bmj.com/site/about/guidelines.xhtml

METHODS

This is a qualitative and quantitative study of an NHS safety improvement programme. We undertook analysis of 2017/2018 Quality Account data from NSCTs in England. We excluded Quality Accounts from ambulance trusts because in 2017/18 they were not required to report. This study has been reported using Standards for Reporting Qualitative Research.[20]

Our objectives were to describe the quality of reporting, and to thematically analyse the reports to derive key learning for the NHS and beyond. We undertook analysis of LfDs as set out in the 2017 amendment to the NHS 2010 Quality Account regulations.

Our evaluation of the quality of reporting involved review of compliance of reports against regulation numbers 27.1 to 27.6 (table 1).[17] Where NSCTs did not fully report we sought to understand why this may have been the case from comments within the Quality Account itself.

Table 1: NHS Quality Accounts LfDs Regulations [17]

Regulation number	Summary of regulatory requirement
**27.1**	The number of patients who have died during the annual reporting period
**27.2**	The number of the deaths (in 27.1) that have undergone a case record review or investigation
**27.3**	An estimate of the number of deaths in 27.2 which the NSCT judges to be more likely than not to have been due to problems in care, with explanation of method to assess this
**27.4**	What the NSCT has learnt from reviews/investigations in relation to deaths (in 27.3)
**27.5**	A description of the actions the NSCT has taken or will take in response to what they have learnt
**27.6**	An assessment of the impact of the actions (from 27.5)

In addition to statutorily required reporting we also looked for evidence within the 2017/18 LfDs report of family/carer engagement, which included evidence of involvement in learning and/or addressing family/carer concerns and/or appointing family liaison officer or similar as a result of a patient death. We also looked for evidence of sharing LfDs incidents both within the NSCT and more widely (for example with other organisations). Both sharing learning and family/carer engagement were recommended in the LfDs national guidance.[15]

Quantitative analysis of regulation 27.1- 27.3 was undertaken and reported using descriptive statistics.

Qualitative analysis to derive key learning themes from regulations 27.4 - 27.6, sharing learning and family/carer engagement was undertaken through document analysis as described by Bowen (2009) using both content and thematic analysis, and through exploratory data analysis. [21, 22] Both deductive and inductive approaches were utilised.

For peer review only

We first identified initial LfDs learning and action themes for reporting, and then developed a classification system for these. The first investigator (ZB) reviewed and analysed twenty 2017/2018 quality accounts, undertook open coding (inductive) and combined this with information presented at the NHS Improvement London LfDs Network (October 2018), where themes (mixed learning and action) from London NSCTs were discussed (deductive). Following the initial review, we reviewed the further 202 NSCT 2017/18 Quality Accounts. Each Account was reviewed by the same reviewer twice to ensure full data capture. Researchers used the process of bracketing to reduce subjective analysis.[23] During data capture further themes emerged, were modified, merged and changed iteratively. Recurring themes were identified using exploratory data analysis,[22] coding, identification of themes, recoding and using frequency charts. Data were captured in Microsoft excel (version 16.15).

Patient and public involvement

This study forms part of a larger programme of work which is overseen by a public and relatives steering group to improve relevance from the perspective of those affected by deaths in healthcare and to reduce biases from the healthcare staff researchers. The steering group have been involved in the planning, design and development of conclusions, through face-to-face meetings and email correspondence. The involvement of a steering group member in authoring this paper has significantly and positively influenced the reporting of this study, ensuring focus on reporting family involvement. The authors reflect that patient and public involvement (PPI) has been essential to this study to ensure that the views of bereaved family members were central to the concerns examined. The reporting of PPI has been undertaken using guidance for reporting involvement of patients and the public 2 – short form (GRIPP2-SF).[24]

For peer review only

RESULTS

Quality Accounts were reviewed for all 222 NSCTs in England.

Quality of Reporting

98 out of 222 (44%) NSCTs reported all six statutory elements of the LfD reporting framework. Two NSCTs did not report any parts of the LfDs regulatory requirements.[25, 26] The total number of deaths reported (regulation 27.1) varied from 3 deaths to 7756 deaths (median 1210.5, range 7753).[27, 28] The number of case record reviews or investigations undertaken relative to the number of patient deaths in individual NSCTs varied between 0.2% and 100% of deaths; the average was 43.7% (median 36.5, range 99.8).

Number of deaths which the NSCT judges to be more likely than not to have been due to problems in care, with explanation of method used to assess this

There was variation between 0 and 13% in the number of deaths which the NSCT judged to be more likely than not to have been due to problems in care (median 0.2, range 13). 22 NSCTs did not report any figure in this section of the quality accounts, reasons given for this included:

- ‘data collection challenges’[29]
- ‘unable to provide a reliable figure’[30]
- ‘we do not carry out investigations with a view to determining whether the death was wholly or partly due to problems in the care provided’[31]
- ‘currently no research base on this for mental health services and no consistent accepted basis for calculating this data’[32]

111 out of 222 NSCTs (50%) noted the use of Structured Judgement Reviews (SJRs) (either Royal College of Physicians or Royal College of Psychiatrists) either alone or in combination with other forms of investigation or review to assess problems in care.[33] NSCTs not using SJRs used a variety of other methods including: Confidential Enquiry into Stillbirths and Deaths in Infancy (CESDI) framework, Root Cause Analysis (RCA) and PReventable Incidents Survival and Mortality (PRISM) methodology.[34, 35]

Plans for assessment of impact

Regulation 26.6 asked NSCTs to undertake ‘an assessment of the impact of the actions’. 105 out of 222 NSCTs (47%) discussed assessment of impact. This includes trusts that had a plan of any sort including a future plan. Several NSCTs used audits and/or quality improvement projects to check that actions are implemented. One NSCT stated ‘Many of these actions are difficult to objectively assess in terms of their impact as they may relate to rare occurrences, which are difficult to meaningfully audit’.[36] The 47% of NSCTs who had a plan for assessment of impact does not include NSCTs that acknowledge the need to assess the impact but stated that it was too early to be able to undertake this (or words to this effect).[37, 38] Some NSCTs have reported the results of the assessment of impact that they have already undertaken.[39] Several NSCTs appear to have misunderstood, for example reiterating the purpose of the LfDs programme, instead of assessing impact.[40, 41]

For peer review only

Evidence involvement of family/carers in learning

In the 2017/18 LfDs reports 37 out of 222 NSCTs (17%) mentioned the involvement of families/carers either in the investigation process or in shared learning or that they communicate with/support/engage/consider families/carers after a patient dies.[42-44] A good example of working with families from one NSCT LfDs report states: 'The Trust continues to learn the importance of communication with families after a death has occurred and that through meaningful engagement after a death by inviting them to contribute to the terms of reference for investigations a more detailed, meaningful and richer account of the person's care and treatment is realised'.[45] One NSCT LfDs report discusses that as an action undertaken they sought to gain better education and training for staff about the importance of positive family engagement through expert external training.[46] 38 NSCTs (17%) discussed as an 'action' that they plan to work with/communicate with/engage/support families/carers. Many of these NSCTs are the same NSCTs already undertaking family/carer engagement.

Evidence learning shared more widely

In the 2017/18 trust LfDs reports 106 out of 222 NSCTs (48%) have shared or plan to share the learning more widely within their own organisation, through a variety of communication mediums: Face to face meetings or events, intranet (as case studies, safety alerts, newsletters).[36, 44, 47] 17 out of 222 (8%) NSCTs have shared or plan to share the learning outside their organisation, with neighbouring NSCTs or other national organisations.[47-50]

Key Findings from the Reports

Lessons learnt

Regulation 27.4 asks NSCTs to describe 'what the provider has learnt from reviews/investigations in relation to deaths' where this was related to deaths which the NSCT judged to be more likely than not to have been due to problems in care (regulation 27.3). 25 out of 222 NSCTs (11%) did not report any lessons learnt from deaths; of these 25 NSCTs, 9 NSCTs had reported 1 or more death judged to be more likely than not due to problems in care, the other 16 NSCTs had either reported zero deaths judged to be more likely than not due to problems in care or had not reported. However, 49 out of 222 NSCTs (22%) which reported that they had no deaths judged more likely than not due to problems in care, also reported lessons learnt, many caveating this with an explanation that they had learnt valuable lessons through the process of case note review/investigation. The most common learning themes from all NSCTs who reported learning can be found in table 2. An overview of the themes arising can be found in the frequency table (figure 1).

Table 2. The five most common learning themes across all NSCTs

Learning Themes	Number of NSCTs citing theme

Poor communication (including language barrier and problems with handover)	90 (46%)
Problem in recognition and escalation of deteriorating patients	83 (42%)
End of life planning or treatment escalation planning not evident/incomplete	82 (42%)
Problems with documentation including consent, details patient team and NOK	80 (41%)
Lack of clinical knowledge, consideration differential/delay diagnosis or seeking advise	53 (27%)

Some NSCTs have undertaken analysis of their learning and described common themes.[51] Some have gone into great detail.[52] Others have described a specific case or cases.[53] Some NSCTs have identified learning and actions together, without differentiating the learning from the action. The lack of structure in reporting makes it difficult to always understand exactly what the problem was leading to the learning.[54] Some NSCTs identified 'Good practice' as learning points.[55] Occasionally NSCTs did not necessarily learn from patient deaths, but from the overall LfDs process.[56]

Actions taken or planned to be taken

NSCTs were asked to undertake 'a description of the actions the NSCT has taken or will take in response to what they have learnt'. 30 out of the 222 NSCTs (14%) did not report any actions taken as a result of learning. One reported that they felt they were 'at too early a stage of development to be able to take actions from specific learning'.[37] The most common action themes from all NSCTs who reported actions can be found in table 3. An overview of the themes arising can be found in the frequency table (figure 2).

Table 3. The five most common action themes across all NSCTs

Action Themes	Number of NSCTs citing theme
Review of process/Standard Operating Procedure (SOP)/pathway	128 (67%)
Highlight guidelines or protocols/policy use of guideline/policies or protocols/treatment bundle/toolkit	96 (50%)
Implementation programme of work/education/bundle	96 (50%)
Quality improvement work or similar	90 (47%)
Work to improve communication/collaboration/shared learning	62 (32%)

The level of detail with regards to actions taken varies greatly with some NSCTs listing some specific actions as bullet points.[39] Others have described a specific case or cases.[57, 58]

For peer review only

DISCUSSION

This study demonstrates wide variation in both the quality of reporting and the findings from LfDs reports. Considering this is a new programme, introduced part-way through 2017/18, with limited guidance, the overall findings are somewhat encouraging. Nearly all NSCTs reported at least one or more element of the statutory LfD requirements. Most NSCTs reported lessons learnt and/or actions taken, while less than half discussed assessment of impact. The lessons learnt were varied. The most common learning theme reported was poor communication, with the most common action theme reported being; review of process/Standard Operating Procedure (SOP)/pathway.

Quality of Reporting

Reporting variation may be due to differences in interpretation of the guidance and statutory requirements. There is no direct financial penalty for a NSCT not reporting some or all elements of the LfDs statutory requirements in their Quality Accounts. However penalties can arise during CQC inspections, when an assessment of implementation of LfDs is carried out.[59, 60]

The different approaches taken by NSCTs and the heterogeneity of data makes comparison difficult. The variation in the percentage of deaths being reviewed/investigated may be due to some NSCTs not having the capacity to review/investigate cases, collect and/or report accurately. NSCTs with a very small number of deaths may find it easier to review all deaths than very large NSCTs. Some NSCTs have had mortality review processes in place for several years and have already been reviewing/investigating deaths, making implementation of the LfDs process easier since the structure for reviewing cases and personnel required are already in place. Some NSCTs may have felt at risk from negative attention by declaring total numbers of deaths and deaths judged more likely than not due to problems in care. Many NSCTs did however report despite the same risk. It is clear from the LfDs reports that several NSCTs, particularly some mental health and community NSCTs, did not feel that the guidance applied to them, however other similar NSCTs were able to comply with reporting. The results could suggest guidance was written with acute NSCTs in mind and perhaps need to be reconsidered for non-acute NSCTs. Similar findings were noted by the CQC in their report ‘Learning from deaths: A review of the first year of NHS trusts implementing the national guidance’.[60]

The variation in deaths judged more likely than not due to problems in care is larger than those noted in previous studies.[2-6] It seems unlikely than many NSCTs would experience no deaths judged more likely than not due to problems in care. This could realistically be the case in specialist NSCTs where the absolute number of total deaths is very small, or community NSCTs with no inpatient beds, but seems unlikely in large acute NSCTs. Despite the improbability several acute NSCTs did report zero deaths judged more likely than not due to problems in care. Further work to understand why these NSCTs reported zero deaths should be undertaken.

The element of the statutory LfDs reporting that prompted poor responses from most NSCTs was ‘An assessment of the impact of the actions’ and describing how they would undertake this. The vast majority of NSCTs have answered this in a vague manner, seemingly through variable interpretation of the regulation. Improvements could be made by issuing further

For peer review only

specific guidance in relation to this element of the reporting. Of the NSCTs who did manage to implement actions and assess impact this was often using quality improvement measurements. The use of quality improvement methodology is felt to be an important overall indicator of quality by the CQC.[61] Guidance on evaluating the impact of interventions is widely available.[62, 63]

Collectively within the LfDs reports, there is much learning, some resulting in impactful actions and high-level organisational learning.[8] This learning could potentially be usefully shared across the NHS and internationally. Some NHS NSCTs appear to have disengaged with the programme. This study suggests a lack of shared learning from the LfDs reports particularly between NSCTs and a lack of family engagement, despite NHS guidance.[19] Since the involvement of families and sharing learning were not statutory requirements of LfDs reporting, they may be underrepresented in the LfDs reports, this should be investigated further before any definite conclusions can be drawn about NSCTs engagement in this element of the LfDs guidance. This study does demonstrate an apparent disparity in organisational learning and safety culture, which results in inequity for families/carers. This should be addressed by the DHSC and associated national bodies. Since the oversight bodies which were established to support the programme in its initial stages have now been stood down this seems unlikely to happen.[64, 65]

Key Findings from the Reports

Overall consistency with regards to identifying, reporting, investigating, learning from deaths in care and taking action has improved across most NSCTs. The continual process of learning, action and reflection which characterises effective organisational learning is essential to ensure the change necessary for safer healthcare. This can only be achieved where information and knowledge affecting patient safety is easily accessible to all members of healthcare staff, supporting an overall safety culture.[66, 67]

Only a small number of NSCTs did not report any learning, suggesting that most NSCTs were able to engage with this aspect of reporting. Many NSCTs have effectively described lessons learnt and actions taken. However, most of the LfDs report recommendations or actions are fairly non-specific; further detail of actions and their measurable impact would be helpful.

It is of concern that the majority of these lessons and recommended actions have previously been identified in national and international reports and inquiries, looking at the problems associated with preventable deaths. Similar problems found in this study are also highlighted in these reports; poor clinical monitoring, poor recognition of the deteriorating patient, diagnostic errors, poor communication, lack of end of life planning, lack of information sharing between services, inadequate drug and fluid management.[68-76] This suggests many of the same problems reoccur and that healthcare systems do not learn from previous failings and adds weight to the proposition that the NHS as a whole cannot become a learning organisation.[77] In view of this, it is reasonable to question whether the learning arising from LfDs reporting will result in meaningful change. If LfDs findings and recommendations are not implemented, systemic redundancy in the initiative is implied. While individual healthcare practitioners do need to take some responsibility, NSCTs and the DHSC should look at systems, such as institutional accountability and LfDs programme oversight to optimise outcomes and minimise the risk of fatal patient safety incidents occurring. This lack of change adds to the growing body of evidence suggesting that traditional approaches to

For peer review only

organisational learning in healthcare, such as learning from when things go wrong (safety-I) have limited effect and may suggest a role for increased learning from the patients who have experienced excellent patient care and outcome despite being seriously unwell (safety-II).[78]

Recommendations

In view of the findings from this study, in order to improve reporting quality, our recommendations are as follows:

- A more structured LfDs reporting template, including all regulatory requirements should be implemented through the Quality Accounts
- NHSE/I specific guidance should be developed on how NSCTs can undertake 'an assessment of the impact of the actions'
- To reinstate LfDs robust regulatory reporting oversight in addition to CQC inspections

In order to improve 'learning and action' from deaths, our recommendations are:

- Annual collection and collation of all NSCT LfDs reporting that is made publicly available
- Further investigation into how NSCTs currently involve bereaved families and carers
- Investment in leadership and support for NHS staff to enable a safety culture

Methodological Limitations

This is an analysis of the very first year of LfDs reporting and reports could underrepresent current NSCT engagement in the LfDs process. NSCTs may be undertaking elements of the LfDs programme that were not statutory reporting requirements such as family/carer engagement, but not reporting on these as it was not a regulatory requirement. NSCT LfDs reports were not created for research analysis and are not standardised, this heterogeneity and subjectivity within the reports reduces equitable comparison.

Despite attempts to minimise inherent researcher bias, such as through PPI involvement and the process of bracketing, the qualitative analysis may have been influenced to a limited extent.

For peer review only

CONCLUSION

Organisations are variably reporting against LfDs regulations, with overall improved consistency in the way that NSCTs identify, report, investigate and learn from deaths in care since the CQC review. [14] However, more could be done to enhance and strengthen the programme impact, and to assess whether LfDs reporting reflects NSCT LfDs engagement.

On the basis of findings from the 2017/18 LfDs reports, national programmes led by multidisciplinary healthcare practitioners should be developed to tackle the most common problems which may have contributed to patient deaths. In the first instance programmes tackling the following issues should be developed or strengthened:

- Improving communication [79]
- Involvement of families in care and in learning [80]
- Processes to share learning (locally and nationally) [81]

Further work is needed to understand which actions taken by NSCTs result in the biggest impact and for this learning to be shared. While LfDs can be difficult and emotive it is fundamental that healthcare systems ensure learning and impactful change occur.

ACKNOWLEDGEMENTS

We would like to acknowledge the work of the Learning from Deaths: Learning and Action (LfDLaA) Public and Relatives steering group in this research. Patient and public involvement in this research was supported by the NIHR UCL Biomedical Research Centre.

For peer review only

REFERENCES

1 World Health Organisation 2019. Patient safety. Accessed July 2020. Available at: https://www.who.int/news-room/fact-sheets/detail/patient-safety
2 Levinson DR 2010. Adverse events in hospitals: national incidence among Medicare beneficiaries. Office of Inspector General for the Department of Health and Human Services. Accessed July 2020. Available at: https://oig.hhs.gov/oei/reports/oei-06-09-00090.pdf
3 Kobewka DM, Van Walraven C et al. The prevelance of potentially preventable deaths in an acute care hospital.: A retrospective cohort. Medicine. 2017 Feb;96(8):e6162. doi:1097/MD.0000000000006162
4 Hogan H, Healey F et al. Preventable deaths due to problems in care in English acute hospitals: retrospective case record review study. BMJ Quality & Safety 2012;21:737-745. doi:10.1136/bmjqs-2011-001159
5 Roberts AP, Morrow G et al. From research to practice: results of 7300 mortality retrospective case record reviews in four acute hospitals in the North-East of England. BMJ Open Qual.2017;6(2):e000123. doi:10.1136/bmjoq-2017-000123
6 Rogne T, Nordseth T et al. Rate of avoidable deaths in a Norwegian hospital trust as judged by retrospective chart review. BMJ Quality & safety. 2019 Jan;28(1):49-55. doi:10.1136/bmjqs-2018-008053
7 NHS Digital 2018. Summary Hospital-level Mortality Indicator (SHMI) - Deaths associated with hospitalisation, England, April 2017 - March 2018. Accessed July 2020. Available at: https://digital.nhs.uk/data-and-information/publications/clinical-indicators/shmi/archive/shmi-april-2017---march-2018
8 Fiol M, Lyles MA. Organizational Learning. The Academy of Management Review. Oct 1985;10(4):803-813
9 Salancik GR, Mendl JR. Corporate attributions as strategic illusions of management control. Administrative Science Quarterly 1984;29:238-254
10 Carroll JS, Edmondson AC. Leading organisational learning in health care. Qual Saf Health Care 2002;11:51–56. doi:10.1136/qhc.11.1.51
11 Mannion R, Braithwaite J. Where is Patient Safety Research and Practice Heading? A Response to Recent Commentaries. Int J Health Policy Manag 2019;8(2):136-137. doi: 10.15171/ijhpm.2018.112
12 Hamblin R, Shuker C Well spotted: but now you need to do something BMJ Quality & Safety Published Online First: 18 September 2020. doi: 10.1136/bmjqs-2019-010659
13 Mazars 2015. Independent review of deaths of people with a Learning Disability or Mental Health problem in contact with Southern Health NHS Foundation Trust April 2011 to March 2015. Accessed July 2020. Available at: https://www.england.nhs.uk/south/wp-content/uploads/sites/6/2015/12/mazars-rep.pdf
14 CQC 2016. Learning, candour and accountability. Accessed July 2020. Available at: https://www.cqc.org.uk/sites/default/files/20161213-learning-candour-accountability-full-report.pdf
15 NQB 2017. National Guidance on Learning from Deaths. Accessed July 2020. Available at: Accessed July 2020. Available at: https://www.england.nhs.uk/wp-content/uploads/2017/03/nqb-national-guidance-learning-from-deaths.pdf
16 NHS Improvement 2017. Implementing the Learning from Deaths framework: key requirements for trust boards. Accessed July 2020. Available at: https://improvement.nhs.uk/documents/1747/170921__Implementing_LfD_-_information_for_boards_JH_amend_3.pdf

For peer review only

17 UK Legislation 2017. The National Health Service (Quality Accounts) (Amendment) Regulations 2017. Accessed July 2020. Available at: http://www.legislation.gov.uk/uksi/2017/744/made

18 NHS 2019. About Quality Accounts. Accessed July 2020. Available at: https://www.nhs.uk/using-the-nhs/about-the-nhs/quality-accounts/about-quality-accounts/

19 NQB 2018. Learning from deaths: Guidance for NHS trusts on working with bereaved families and carers. Accessed Sept 2020. Available at: https://www.england.nhs.uk/wp-content/uploads/2018/08/learning-from-deaths-working-with-families-v2.pdf

20 O'Brien BC, Harris IB et al. Standards for reporting qualitative research: a synthesis of recommendations. Acad Med. 2014;89(9):1245-1251. doi:10.1097/ACM.0000000000000388

21 Bowen GA. 2009. Document Analysis as a Qualitative Research Method, Qualitative Research Journal. 9 (2). 27-40. DOI:10.3316/QRJ0902027

22 Stebbins R. Exploratory Data Analysis. In: Given LM, The Sage Encyclopedia of Qualitative Research Methods, Vol. 1, Ed. 1. Sage 2008:325-326

23 Tufford L, Newman P. Bracketing in Qualitative Research. Qualitative Social Work 2010;11:80-96. doi:10.1177/1473325010368316

24 Staniszewska S, Simera I et al. GRIPP2 reporting checklists: tools to improve reporting of patient and public involvement in research. BMJ 2017;358:j3453. doi: 10.1136/bmj.j3453

25 Worcestershire Acute Hospitals NHS Trust Quality Account 2017/18. Accessed July 2020. Available at: https://www.worcsacute.nhs.uk/stoma-care/2146-quality-account-2017-18/file

26 North Staffordshire Combined Healthcare NHS Trust Quality Account 2017/18. Accessed July 2020. Available at: http://389w1t43oria4ctlpf36b5lz-wpengine.netdna-ssl.com/wp-content/uploads/2018/10/Combined-Quality-Account-2017-18.pdf

27 Moorfields Eye Hospital NHS Foundation Trust Quality Account 2017/18. Accessed July 2020. Available at: https://www.moorfields.nhs.uk/sites/default/files/Moorfields%20annual%20report%202017-18.pdf

28 North East London Foundation Trust Quality Account 2017/18. Accessed July 2020. Available at: https://www.nelft.nhs.uk/download.cfm?doc=docm93jijm4n3126.pdf&ver=5090

29 North Middlesex University Hospital NHS Trust Quality Account 2017/18. Accessed July 2020. Available at: http://www.northmid.nhs.uk/Portals/0/201809%20-%20NMUH%20Final%20Quality%20Account%202017-18.pdf?ver=2018-09-18-125322-917

30 Royal Devon and Exeter NHS Foundation Trust Quality Account 2017/18. Accessed July 2020. Available at: https://www.rdehospital.nhs.uk/documents/trust/documents/annual-report-2017-2018.pdf

31 Black Country Partnership NHS Foundation Trust Quality Account 2017/18. Accessed July 2020. Available at: https://www.bcpft.nhs.uk/documents/about-us/key-documents/annual-report/1851-bcp-annual-report-2018/file

32 Sheffield Health and Social Care NHS Foundation Trust Quality Account 2017/18. Accessed July 2020. Available at: https://www.shsc.nhs.uk/sites/default/files/2019-12/Quality%20Report%202017-2018.pdf

33 Healthcare Quality Improvement Partnership 2018. Royal College of Physicians: National mortality case record review. Annual report. Accessed July 2020. Available at: https://www.rcplondon.ac.uk/projects/outputs/national-mortality-case-record-review-nmcrr-annual-report-2018

34 MBRRACE-UK 2017. Perinatal Confidential Enquiry. Confidential Enquiry into Stillbirths and Deaths in Infancy (CESDI) framework:19-20. Accessed July 2020. Available

at: https://www.npeu.ox.ac.uk/downloads/files/mbrrace-uk/reports/MBRRACE-UK%20Intrapartum%20Confidential%20Enquiry%20Report%202017%20-%20final%20version.pdf
35 Hogan H 2014. Preventable, Incidents, Survival and Mortality Study 2 (PRISM) Medical Record Review Manual. Accessed July 2020. Available at: https://improvement.nhs.uk/documents/1423/PRISM_2_Manual_V2_Jan_14.pdf
36 University Hospital Southampton NHS Foundation Trust Quality Account 2017/18. Accessed July 2020. Available at: https://www.uhs.nhs.uk/Media/SUHTInternet/AboutUs/AnnualReportsStrategiesandPlans/201718/Annual-report-2017-18.pdf
37 Royal United Hospitals Bath NHS Foundation Trust. Accessed July 2020. Available at: https://www.ruh.nhs.uk/about/annual_report/documents/Annual_Report_2017-18.pdf
38 Great Western Hospitals NHS Foundation Trust Quality Account 2017/18. Accessed July 2020. Available at: https://www.gwh.nhs.uk/media/309191/gwhnhsftannualqualityaccounts2017-18.pdf
39 Salisbury NHS Foundation Trust Quality Account 2017/18. Accessed July 2020. Available at: https://www.salisbury.nhs.uk/AboutUs/TrustReportsAndReviews/Documents/SalisburyNHSFoundationTrustQualityAccount2017_2018.pdf
40 Gloucestershire Hospitals NHS Foundation Trust Quality Account 2017/18. Accessed July 2020. Available at: https://www.gloshospitals.nhs.uk/media/documents/QA-A4-17-18.pdf
41 Isle of Wight NHS Trust Quality Account 2017/18. Accessed July 2020. Available at: https://www.iow.nhs.uk/Downloads/Quality%20Account/Quality%20Accout%202017-18.pdf
42 Tameside and Glossop Integrated Care NHS Foundation Trust Quality Account 2017/18. Accessed July 2020. Available at: https://www.tamesidehospital.nhs.uk/Documents/AnnualReport20172018.pdf
43 Buckinghamshire Healthcare NHS Trust Quality Account 2017/18. Accessed July 2020. Available at: https://www.buckshealthcare.nhs.uk/Downloads/quality-account/08%20Quality%20Accounts_2017_18.pdf
44 Birmingham Women's and Children's NHS Foundation Trust Quality Account 2017/18. Accessed July 2020. Available at: https://bwc.nhs.uk/download.cfm?doc=docm93jijm4n2490.pdf&ver=3456
45 Pennine Care NHS Foundation Trust Quality Account 2017/18. Accessed July 2020. Available at: https://www.penninecare.nhs.uk/application/files/4615/6172/0636/Quality_account_2017-18.pdf
46 South Staffordshire and Shropshire Healthcare NHS Foundation Trust Quality Account 2017/18. Accessed July 2020. Available at: https://improvement.nhs.uk/documents/4294/South_Staffordshire_Shropshire_Healthcare_Annual_Report_and_Accounts_2017-18.PDF
47 Royal Free London NHS Foundation Trust Quality Account 2017/18. Accessed July 2020. Available at: https://www.royalfree.nhs.uk/uploads/RF_quality_report_2017-18.pdf
48 South west Yorkshire Partnership NHS Foundation Trust Quality Account 2017/18. Accessed July 2020. Available at: https://www.southwestyorkshire.nhs.uk/wp-content/uploads/2018/08/SWYPT-annual-report-and-accounts-2017-18-FINAL.pdf
49 Northumbria Healthcare NHS Foundation Trust Quality Account 2017/18. Accessed July 2020. Available at: https://www.northumbria.nhs.uk/wp-content/uploads/2018/07/FINAL-NHFT-ARA-2017-18.pdf

For peer review only

50 Greater Manchester Mental Health NHS Foundation Trust Quality Account 2017/18. Accessed July 2020. Available at: https://www.gmmh.nhs.uk/download.cfm?doc=docm93jijm4n3649.pdf&ver=7138
51 The Christie NHS Foundation Trust Quality Account 2017/18. Accessed July 2020. Available at: https://www.christie.nhs.uk/media/5682/the-christie-nhs-ft-quality-report-2017-18.pdf
52 Northumberland, Tyne & Wear NHS Foundation Trust Quality Account 2017/18. Accessed July 2020. Available at: https://improvement.nhs.uk/documents/4419/Northumberland_Tyne___Wear_NHS_FT_Annual_Report_and_Accounts_2017-18.PDF
53 The Walton Centre NHS Foundation Trust Quality Account 2017/18. Accessed July 2020. Available at: https://www.thewaltoncentre.nhs.uk/uploadedfiles/THE%20WALTON%20CENTRE%20FINAL%20MERGED%20ANNUAL%20REPORT%20ACCOUNTS%20QUALITY%20ACCOUNT%202017_18.pdf
54 Kent & Medway NHS and Social Care Partnership Trust Quality Account 2017/18. Accessed July 2020. Available at: https://www.kmpt.nhs.uk/media/1234/quality-account-17-18.pdf
55 Bradford Teaching Hospitals NHS Foundation Trust Quality Account 2017/18. Accessed July 2020. Available at: https://www.bradfordhospitals.nhs.uk/wp-content/uploads/2018/07/Annual-Report-and-Accounts-2017-18.pdf
56 Maidstone and Tunbridge Wells NHS Trust Quality Accounts 2017/18. Accessed July 2020. Available at: https://www.mtw.nhs.uk/wp-content/uploads/2018/06/MTW-Quality-Accounts-2018.pdf
57 Cambridge University Hospitals NHS Foundation Trust Quality Account 2017/18. Accessed July 2020. Available at: https://www.cuh.nhs.uk/sites/default/files/Annual%20Report%20and%20Accounts%20201718.pdf
58 Camden and Islington NHS Foundation Trust Quality Account 2017/18. Accessed July 2020. Available at: https://www.cuh.nhs.uk/sites/default/files/Annual%20Report%20and%20Accounts%20201718.pdf
59 CQC 2018. Inspection framework: NHS trusts and foundation trusts. Trust-wide well-led. Accessed July 2020. Available at: https://www.cqc.org.uk/sites/default/files/20200115_Trust_wide_well_led_inspection_framework_V7.pdf
60 CQC 2019. Learning from deaths: A review of the first year of NHS trusts implementing the national guidance Accessed July 2020. Available at: https://www.cqc.org.uk/sites/default/files/20190315-LfD-Driving-Improvement-report-FINAL.pdf
61 CQC 2018. Quality improvement in hospital trusts. Accessed July 2020. Available at: https://www.cqc.org.uk/sites/default/files/20180911_QI_hospitals_FINAL.pdf
62 NHS 2015. How to understand and measure impact. Accessed Sept 2020. Available at: https://www.england.nhs.uk/wp-content/uploads/2015/06/bcf-user-guide-04.pdf.pdf
63 Clarke Geraldine M, Conti Stefano et al. Evaluating the impact of healthcare interventions using routine data BMJ 2019;365:l2239. doi: 10.1136/bmj.I2239
64 RCP 2019. National Mortality Case Record Review Programme. Accessed July 2020. Available at: https://www.rcplondon.ac.uk/projects/national-mortality-case-record-review-programme

65* Ocloo J, Smith D. Bereaved families have been used as pawns in a political game. Health Service Journal. February 2021. Accessed February 2021. Available at: https://www.hsj.co.uk/patient-safety/bereaved-families-have-been-used-as-pawns-in-a-political-game/7029561.article
65 [66]Carroll JS, Edmondson AC. Leading organisational learning in healthcare. BMJ Quality & Safety 2002;11(1):51-6. doi: 10.1136/qhc.11.1.51
66 [67]Mannion R, Konteh FH, Davies HTO. Assessing organisational culture for quality and safety improvement: a national survey of tools and tool use. BMJ Quality & Safety 2009;153:153-6. doi: 10.1136/qshc.2007.024075
67 [68]National Confidential Enquiry into Patient Outcomes and Deaths (NCEPOD) 2018. Themes and recommendations common to all hospital specialties. Accessed July 2020. Available at: https://www.ncepod.org.uk/CommonThemes.pdf
68 [69]NHS England 2014. Patient safety alert. Stage three: directive. Improving medication error incident reporting and learning. Accessed July 2020. Available at: https://www.england.nhs.uk/wp-content/uploads/2014/03/psa-sup-info-med-error.pdf
69 [70]Singh H, Schiff GD, Graber ML, Onakpoya I, Thompson MJ. The global burden of diagnostic errors in primary care. BMJ Qual Saf 2017;26:484-494. doi: 10.1136/bmjqs-2016-005401
70 [71]Oates A. Learning from suicide-related claims. A thematic review of NHS Resolution data. NHS Resolution. September 2018. Accessed July 2020. Available at: https://resolution.nhs.uk/wp-content/uploads/2018/09/NHS-Resolution_learing_from_suicide_claims_148pp_ONLINE1.pdf
71 [72]Government of Western Australia, Department of Health 2019. From death we learn 2018. Accessed July 2020. Available at: https://ww2.health.wa.gov.au/~/media/Files/Corporate/Reports%20and%20publications/PDF/From-Death-We-Learn-2018.pdf
72 [73]PHSO 2014. An avoidable death of a three-year-old child from sepsis. Accessed July 2020. Available at: https://www.ombudsman.org.uk/sites/default/files/An_avoidable_death_of_a_three_year_old_child_from_sepsis%20_report.pdf
73 [74]Francis R. Report of the Mid Staffordshire NHS Foundation Trust Public Inquiry: Executive summary. Department of Health, 2013. Accessed July 2020. Available at: https://assets.publishing.service.gov.uk/government/uploads/system/uploads/attachment_data/file/279124/0947.pdf
74 [75]Kirkup B. The Report of the Morecambe Bay Investigation. Department of Health, 2015. Accessed July 2020. Available at: https://assets.publishing.service.gov.uk/government/uploads/system/uploads/attachment_data/file/408480/47487_MBI_Accessible_v0.1.pdf
75 [76]Walshe K, Higgins J. The use and impact of inquiries in the NHS. BMJ 2002;325(7369): 895–900. doi: 10.1136/bmj.325.7369.895
76 [77]Sheaff R, Pilgrim D. Can learning organisations survive in the newer NHS? Implementation science 2006:1(27). doi: 10.1186/1748-5908-1-27
77 [78]Sujan M. A Safety-II perspective on organisational learning in healthcare organisations. Int J Health Policy Manag 2018;7(7):662-666. doi: 10.15171/ijhpm.2018.16
79 NHS England 2020. The NHS Engagement and Communications Development Programme. Accessed February 2021. Available at: https://www.england.nhs.uk/nhs-communications-development/
80 Making Families Count. Accessed February 2021. Available at: https://www.makingfamiliescount.org.uk

For peer review only

81 NHS Education for Scotland. The Knowledge Network. Accessed February 2021. Available at: http://www.knowledge.scot.nhs.uk/adverse-events/sharing-learning.aspx

For peer review only

Footnotes

Contributors

ZB conceived and designed the study, undertook analysis, interpretation of the data and drafted the manuscript.
CVP provided critical input to the design, analysis, interpretation of the data and revised the manuscript critically.
DB provided critical input to the design, analysis, interpretation of the data and revised the manuscript critically.
SRM provided critical input to the design, analysis, interpretation of the data and revised the manuscript critically.
The corresponding author attests that all listed authors meet authorship criteria and that no others meeting the criteria have been omitted. ZB is the guarantor.

Funding

This study was supported by the National Institute of Health Research (NIHR) University College London (UCL) Biomedical Research Centre Patient and Public Involvement starter funding (BRC617/PPI/ZB/104990).

Competing interests

ZB worked at NHS Improvement in the medical directorate from August 2017 to August 2018, during which time she undertook some work on the LfDs programme. SRM is the National Clinical Director for Critical and Perioperative Care for NHS England/NHS Improvement, SRM has no link with the LfDs programme. CVP and DB do not have any competing interests to declare. All authors have completed the ICMJE uniform disclosure form at www.icmje.org/coi_disclosure.pdf

Ethical approval

This study is an analysis of publicly available data and does not require ethical approval.

Data availability statement

All the data for this study is publicly available from the 2017/18 NHS Quality Accounts.

For peer review only

Figure Legends

Figure 1. Frequency table of lessons learnt (all NSCTs n=222)

A – Problem in recognition & escalation of deteriorating patients
B – Lack of or awareness of or following protocol/guideline/bundle
C – Problem in assessment or experience related to learning disabilities
D – Poor communication (including language barrier & problems with handover)
E – Problem with end-of-life planning or treatment escalation planning
F – Problem with death certification or confirming death
G – Problem with discharge (timing/letters/delay/information for patients)
H – Difficulty accessing support services/ Out of Hours services/ Specialist services
I – Problem/lack of risk assessment/interventions
J – Lack of knowledge of hospital layout/equipment
K – Problem with patient transfers
L – Problem assessing/providing nutrition/fluids/electrolytes
M – Lack of senior/consultant review, input, planning
N – Excellent/good care/management
O – Prompt senior review
P – Good communication/collaboration/teamwork
Q – Lack of clinical knowledge, consideration differential diagnosis or seeking advise
R – Problem with/lack of prescribing or side-effects or administration of medications
S – Problem with 'Duty of Candour'
T – Delay to acting on results
U – Problems with documentation including consent
V – Delay/problem in requesting or interpretation of investigations
W – Lack of/problem with monitoring/observations/recording
X - Lack of/or problem with sharing information with other providers/services/specialties
Y – Delay in reviewing patient
Z – Delay in treatment/incomplete management including care plans and pain management
AA – Poor continuity of care/team work
AB – Concerns with pre-hospital care (residential settings/wider societal issues)
AC – Lack of familiarity with or standardisation or availability of equipment
AD – Problem related to workforce or staffing or supervision of staff
AE – Misfiled documents/lost notes/problems in storage or access of notes/scans
AF – Problem with recognition/management of Acute Kidney Injury
AG – Lack of Multidisciplinary Team involvement/discussion/decision
AH – Problem with competency or complication in undertaking procedure/operation
AI – Problem related to infection control
AJ – Lack of/problem with assessment of mental health needs and/or follow-up
AK – Problem related to appropriateness of patient ward allocation or relocation
AL – Problem with pre-op assessment/peri-op management
AM – Problem with capacity/flow/hospital of department pressures (including A&E)
AN – Deviation from treatment plan or plan not linked with clinical record
AO - Follow-up planning not evident or incomplete/problem with follow-up
AP – Problem related to management of physical health problem in mental health setting
AQ – Problem due to patient not wanting to/unable to engage with treatment (with capacity)
AR – Problem after death (related to post-mortem/forensic services or investigation)
AS – Problem with the recognition/management of drug/alcohol withdrawal/recovery
AT – Lack of supervision or safe accommodation for vulnerable patient

For peer review only

AU – Lack of/problem with engagement with/support of families/carers

Figure 2. Frequency table of actions taken (all NSCTs n=222)

A – Work to improve communication/collaboration/shared learning
B – Improved end-of-life planning (including communication)
C – Improved effectiveness of handover
D– Highlight or new or use of guidelines/protocols/policy/protocol/treatment bundle/toolkits
E – Improved mortality review process
F – Undertake or improve risk assessment/governance process/reporting system
G – Review of process/SOP/pathway/audit process
H – Quality improvement work or similar
I – 'Raising awareness' or 'Importance of' or 'reflecting on' (not qualified)
J – Implementation of a programme of work or education (including simulation & induction)
K – Raising awareness (with specific example – 'nursing dashboard', 'case presentation')
L – Use of technology (for example electronic recording of observations)
M – Rota adjusted to provide better cover or extra lists/sessions
N – Working/communicating with/supporting families (not end-of-life planning)
O – 'More effective', 'continued efforts', 'seeking advice' 'review/introduce' (not qualified)
P – Solution involving medical examiner role
Q – Improved senior/consultant involvement (with specific examples)
R – External or internal (peer review) mortality/governance review or investigation
S – Identification of high-risk patients early
T – Extend post-op recovery monitoring
U – Improved documentation/coding
V – Follow-up of action plans
W – Plan to improve sharing of learning
X – Ensure Early Warning System in place/utilised correctly
Y – Improvement of results reporting & acknowledgement process/archiving results/scans
Z – Multidisciplinary team/programme of work set-up to address specific problem
AA – Seek-out and follow expert advise
AB – Improve review methodology (Such as Structured Judgement Review training)
AC – Develop regional Learning from Deaths network or similar
AD – Negotiate with coroner for earlier post-mortem reports
AE – Increase emergency operating capability (additional emergency theatre availability)
AF – Improvement to bereavement facilities
AG – Improved infection control measures
AH – Supervision discussions/support/feedback for those involved in incidents
AI – Improved cross-specialty collaboration
AJ – Increased specialist equipment availability or specialist teams or specialist roles
AK – Increased engagement in LeDeR process

For peer review only

Figure 1. Frequency table of lessons learnt (all NSCTs n=222)

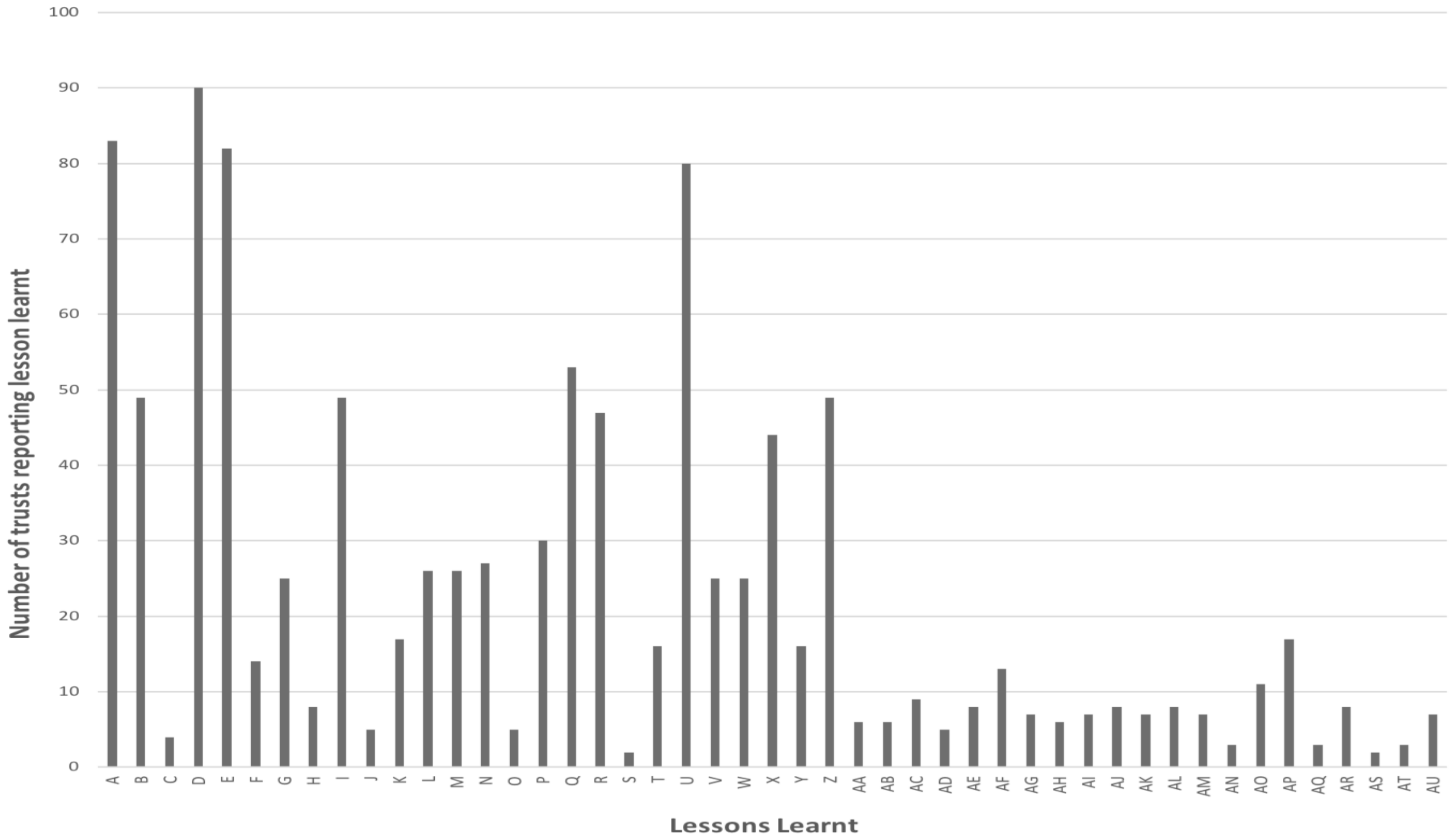

Figure 2. Frequency table of actions taken (all NSCTs n=222)

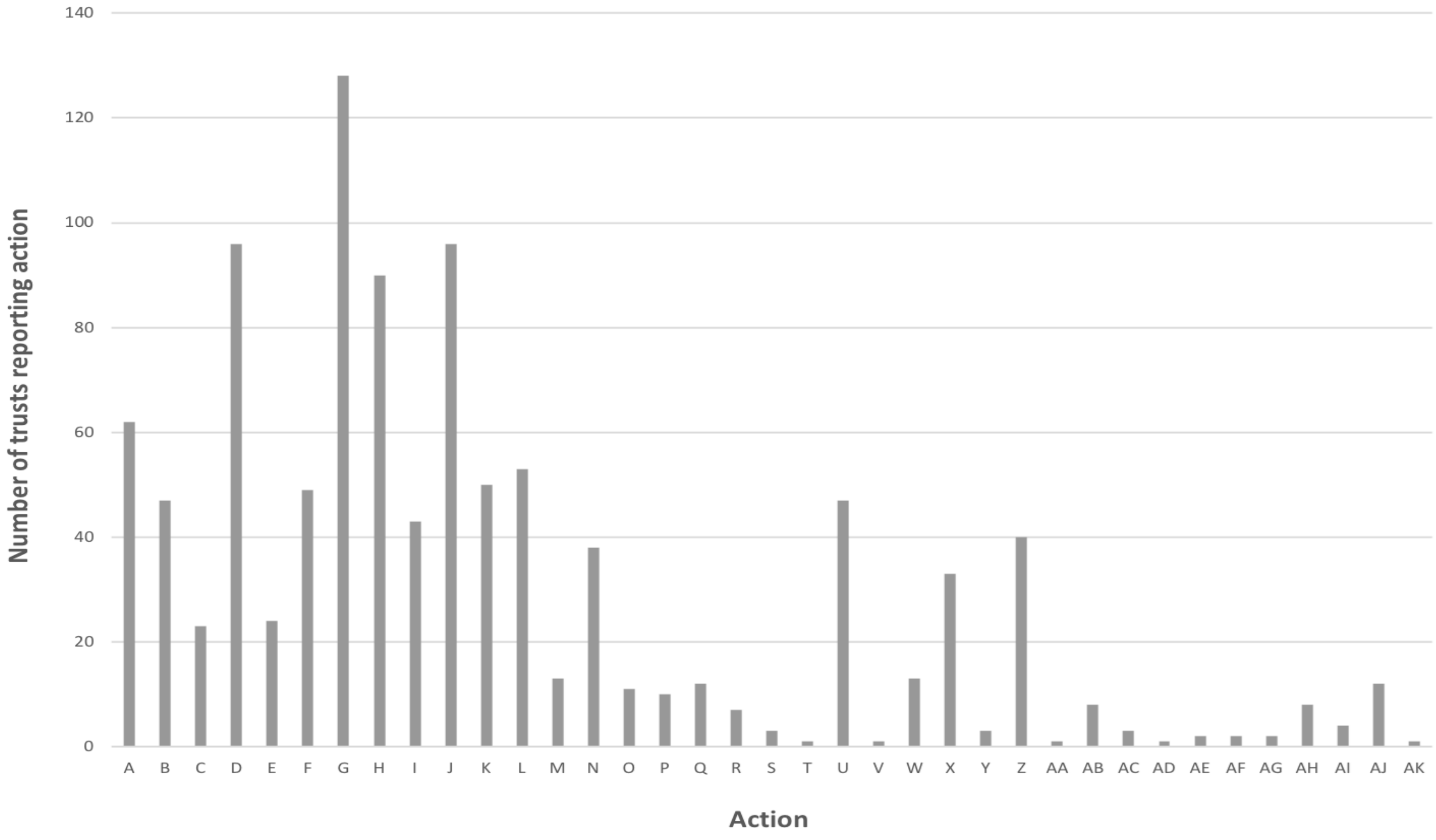

Standards for Reporting Qualitative Research (SRQR)

O'Brien BC, Harris IB et al. Standards for reporting qualitative research: a synthesis of recommendations. Acad Med. 2014;89(9):1245-1251. doi:10.1097/ACM.0000000000000388

No.	Topic	Item	Page number and line in 030321.BMJOpen_LfDs where relevant information can be found
**Title and abstract**			
**S1**	Title	Concise description of the nature and topic of the study Identifying the study as qualitative or indicating the approach (e.g., ethnography, grounded theory) or data collection methods (e.g., interview, focus group) is recommended	Page 1, lines 1-3
**S2**	Abstract	Summary of key elements of the study using the abstract format of the intended publication; typically includes background, purpose, methods, results, and conclusions	Page 2
**Introduction**			
**S3**	Problem formulation	Description and significance of the problem/phenomenon studied; review of relevant theory and empirical work; problem statement	Page 4, lines 3 - 10 and lines 23 - 40
**S4**	Purpose or research question	Purpose of the study and specific objectives or questions	Page 4, lines 49 and 50<br>Page 5, lines 1 and 2
**Methods**			
**S5**	Qualitative approach and research paradigm	Qualitative approach (e.g., ethnography, grounded theory, case study, phenomenology, narrative research) and guiding theory if appropriate; identifying the research paradigm (e.g., postpositivist, constructivist/interpretivist) is also recommended; rationale	Qualitative approach described:<br>Page 6, lines 32 - 35<br>Page 7, lines 1 - 11<br><br>Research paradigm not appropriate in this study.
**S6**	Researcher characteristics and reflexivity	Researchers' characteristics that may influence the research, including personal attributes, qualifications/experience, relationship with participants, assumptions, and/or presuppositions; potential or	Page 7, line 8<br>Page 21, lines 24 - 29

For peer review only

		actual interaction between researchers' characteristics and the research questions, approach, methods, results, and/or transferability	
**S7**	Context	Setting/site and salient contextual factors; rationale	Page 6, lines 4 - 6
**S8**	Sampling strategy	How and why research participants, documents, or events were selected; criteria for deciding when no further sampling was necessary (e.g., sampling saturation); rationale	Page 6, lines 4 - 6
**S9**	Ethical issues pertaining to human subjects	Documentation of approval by an appropriate ethics review board and participant consent, or explanation for lack thereof; other confidentiality and data security issues	Page 21, line 33
**S10**	Data collection methods	Types of data collected; details of data collection procedures including (as appropriate) start and stop dates of data collection and analysis, iterative process, triangulation of sources/methods, and modification of procedures in response to evolving study findings; rationale	Page 6, lines 4 – 7, lines 13 – 16, table 1, lines 22 – 27, lines 29 – 30, lines 32 – 35<br>Page 7, lines 1 - 11
**S11**	Data collection instruments and technologies	Description of instruments (e.g., interview guides, questionnaires) and devices (e.g., audio recorders) used for data collection; if/how the instrument(s) changed over the course of the study	Page 7, line 11
**S12**	Units of study	Number and relevant characteristics of participants, documents, or events included in the study; level of participation (could be reported in results)	Page 4, lines 37 - 40<br>Page 6, lines 4 – 6, lines 13 – 16, table 1, lines 22 - 27<br>Page 8, line 3, line 8
**S13**	Data processing	Methods for processing data prior to and during analysis, including transcription, data entry, data management and security, verification of data integrity, data coding, and anonymization/deidentification of excerpts	Page 6, lines 29 – 30, lines 32 – 35<br>Page 7, lines 1 - 11
**S14**	Data analysis	Process by which inferences, themes, etc., were identified and developed, including the researchers involved in data analysis; usually references a specific paradigm or approach; rationale	Page 6, lines 32 – 35<br>Page 7, lines 1 - 11

For peer review only

**S15**	Techniques to enhance trustworthiness	Techniques to enhance trustworthiness and credibility of data analysis (e.g., member checking, audit trail, triangulation); rationale	Page 7, lines 2 – 6, line 7, line 8, lines 15 - 24
**Results/findings**			
**S16**	Synthesis and interpretation	Main findings (e.g., interpretations, inferences, and themes); might include development of a theory or model, or integration with prior research or theory	Page 8, lines 3 - 47<br>Page 9, lines 4 - 46, table 2<br>Page 10, table 3, lines 2 - 23<br>Figure 1 and figure 2
**S17**	Links to empirical data	Evidence (e.g., quotes, field notes, text excerpts, photographs) to substantiate analytic findings	Page 8, lines 21 - 26, lines 40 - 42<br>Page 9, lines 7 – 11
**Discussion**			
**S18**	Integration with prior work, implications, transferability, and contribution(s) to the field	Short summary of main findings; explanation of how findings and conclusions connect to, support, elaborate on, or challenge conclusions of earlier scholarship; discussion of scope of application/generalizability; identification of unique contribution(s) to scholarship in a discipline or field	Page 11, lines 3 – 10, lines 33 – 35, lines 37 - 44<br>Page 12, lines 6 – 8, lines 35 – 43, lines 48 – 50<br>Page 13, lines 1 - 2
**S19**	Limitations	Trustworthiness and limitations of findings	Page 3, lines 5 - 22
**Other**			
**S20**	Conflicts of interest	Potential sources of influence or perceived influence on study conduct and conclusions; how these were managed	Page 21, lines 24 - 29
**S21**	Funding	Sources of funding and other support; role of funders in data collection, interpretation, and reporting	Page 21, lines 18 - 20

For peer review only

BMJ Open

NHS 'Learning from Deaths' reports: A qualitative and quantitative document analysis of the first year of a countrywide patient safety programme

Journal:	*BMJ Open*
Manuscript ID	bmjopen-2020-046619.R2
Article Type:	Original research
Date Submitted by the Author:	16-Jun-2021
Complete List of Authors:	Brummell, Zoe; University College London, Centre for Perioperative Medicine, Department of Targeted Intervention, Division of Surgery and Interventional Science; University College London Hospitals NHS Foundation Trust, Department of Anaesthesia and Intensive Care Medicine<br>Vindrola-Padros, Cecilia; University College London, Department of Targeted Intervention; UCL, UCL<br>Braun, Dorit; Advisor/Lived experience<br>Moonesinghe, S. Ramani; UCL/UCLH Surgical Outcomes Research Centre, Centre for Perioperative Medicine, Department for Targeted Intervention, Division of Surgery and Interventional Science; Health Services Research Centre, , National Institute for Academic Anaesthesia, Royal College of Anaesthetists
**Primary Subject Heading**:	Health policy
Secondary Subject Heading:	Health services research, Medical management, Qualitative research
Keywords:	Health & safety < HEALTH SERVICES ADMINISTRATION & MANAGEMENT, Health policy < HEALTH SERVICES ADMINISTRATION & MANAGEMENT, Organisational development < HEALTH SERVICES ADMINISTRATION & MANAGEMENT, Quality in health care < HEALTH SERVICES ADMINISTRATION & MANAGEMENT, QUALITATIVE RESEARCH

SCHOLARONE™ Manuscripts

I, the Submitting Author has the right to grant and does grant on behalf of all authors of the Work (as defined in the below author licence), an exclusive licence and/or a non-exclusive licence for contributions from authors who are: i) UK Crown employees; ii) where BMJ has agreed a CC-BY licence shall apply, and/or iii) in accordance with the terms applicable for US Federal Government officers or employees acting as part of their official duties; on a worldwide, perpetual, irrevocable, royalty-free basis to BMJ Publishing Group Ltd ("BMJ") its licensees and where the relevant Journal is co-owned by BMJ to the co-owners of the Journal, to publish the Work in this journal and any other BMJ products and to exploit all rights, as set out in our licence.

The Submitting Author accepts and understands that any supply made under these terms is made by BMJ to the Submitting Author unless you are acting as an employee on behalf of your employer or a postgraduate student of an affiliated institution which is paying any applicable article publishing charge ("APC") for Open Access articles. Where the Submitting Author wishes to make the Work available on an Open Access basis (and intends to pay the relevant APC), the terms of reuse of such Open Access shall be governed by a Creative Commons licence – details of these licences and which Creative Commons licence will apply to this Work are set out in our licence referred to above.

Other than as permitted in any relevant BMJ Author's Self Archiving Policies, I confirm this Work has not been accepted for publication elsewhere, is not being considered for publication elsewhere and does not duplicate material already published. I confirm all authors consent to publication of this Work and authorise the granting of this licence.

For peer review only

NHS 'Learning from Deaths' reports: A qualitative and quantitative document analysis of the first year of a countrywide patient safety programme

Authors:

Zoe Brummell (0000-0002-6296-5049), Researcher/Clinician, Department of Anaesthesia, University College London Hospitals NHS Foundation Trust, London, NW1 2BU

Cecilia Vindrola-Padros (0000-0001-7859-1646), Senior Researcher, Department of Targeted Intervention, University College London, Charles Bell House, 43-45 Foley Street, London, W1W 7TS

Dorit Braun, Advisor/Lived experience, Learning from Deaths: Learning and Action Public and Relatives Steering Group member

S. Ramani Moonesinghe (0000-0002-6730-5824), Professor, Centre for Perioperative Medicine, Department for Targeted Intervention, University College London, Charles Bell House, 43-45 Foley Street, London, W1W 7TS

Corresponding author:

Zoe Brummell (0000-0002-6296-5049), Researcher/Clinician, Department of Anaesthesia, University College London Hospitals NHS Foundation Trust, London, England, NW1 2BU

z.brummell@nhs.net

For peer review only

ABSTRACT

Objectives
To review how NHS Secondary Care Trusts (NSCTs) are using the Learning from Deaths (LfDs) programme to learn from and prevent, potentially preventable deaths.

Introduction
Potentially preventable deaths occur worldwide within healthcare organisations. In England, inconsistencies in how NSCTs reviewed, investigated and shared learning from deaths, resulted in the introduction of national guidance on 'Learning from Deaths' in 2017. This guidance provides a 'framework for identifying, reporting, investigating and learning from deaths'. Amendments to NHS Quality Account regulations, legally require NSCTs in England to report quantitative and qualitative information relating to patient deaths annually. The programme intended NSCTs would share this learning and take measurable action to prevent future deaths.

Method
We undertook qualitative and quantitative secondary data, document analysis of all NSCTs LfDs reports within their 2017/18 Quality Accounts (n=222).

Results
All statutory elements of LfDs reporting were reported by 98 out of 222 (44%) NSCTs. The percentage of deaths judged more likely than not due to problems in healthcare was between 0% and 13%. The majority of NSCTs (89%) reported lessons learnt; the most common learning theme was poor communication. 106 out of 222 NSCTs (48%) have shared or plan to share the learning within their own organisation. The majority of NSCTs (86%) reported actions taken and 47% discussed or had a plan for assessment of impact. 37 out of 222 NSCTs (17%) mentioned involvement of bereaved families.

Conclusions
The wide variation in reporting demonstrates that some NSCTs have engaged fully with LfDs, while other NSCTs appear to have disengaged with the programme. This may reveal a disparity in organisational learning and patient safety culture which could result in inequity for bereaved families. Many themes identified from the LfDs reports have previously been identified by national and international reports and inquiries.

For peer review only

STRENGTHS AND LIMITATIONS OF THIS STUDY

This is the first study to our knowledge to analyse Learning from Deaths (LfDs) reporting.

Quality Accounts from all NSCTs in England legally required to report LfDs were included in both the quantitative and qualitative analysis. Including all NSCTs in the qualitative analysis has ensured complete and thorough data capture. Despite attempts to minimise inherent researcher bias, the qualitative analysis may have been influenced by to some extent.

This study has ensured the inclusion of views from bereaved relatives, through Patient and Public Involvement. The authors conclude that these views are essential to improving patient safety.

This is an analysis of the very first year of LfDs reporting and reports could underrepresent current NSCT engagement in the LfDs process.

NSCTs may be undertaking elements of the LfDs programme that were not statutory reporting requirements such as family/carer engagement, but not reporting on these as it was not a regulatory requirement.

For peer review only

INTRODUCTION

Globally, adverse events while receiving healthcare is a leading cause of morbidity and mortality.[1] The percentage of preventable or potentially preventable deaths is likely to lie somewhere between 0.5% and 8.4% of hospital deaths.[2-6] In England between April 2017 and end of March 2018 there were 299,000 deaths occurring in hospital or within 30 days of discharge, this amounts to an estimate of between 1,495 and 25,116 potentially preventable deaths.[7] There is a moral imperative for healthcare organisations to learn from these deaths and take measurable action to prevent potentially preventable deaths.

Healthcare organisations are made up of individuals who have the ability to learn: however organisational learning is 'more than the sum of individual learning' and is distinct from unreflective action taking.[8] It is more than simply creating change for change's sake, as an 'illusion of learning'.[9] Organisational learning is the ability to apply knowledge and understanding to increase effective organisational action.[8, 10] Effective organisational learning is crucial to improve patient safety and probably requires both safety-I (understanding why things go wrong) and safety-II (understanding why things go right) approaches.[11, 12] Central regulation and performance management may have some effect on improving care, but quality improvement, leadership, public engagement, proper resourcing, education, and training are needed for a safer health service.[12]

In April 2016 an independent review demonstrated a lack of systematic approach and meaningful change in response to unexpected deaths at Southern Health NHS Foundation Trust.[13] The Care Quality Commission (CQC), which is responsible for monitoring, inspection and regulation of healthcare services within England, conducted a wider review into the investigations of deaths. They found inconsistencies in the way NHS Secondary Care Trusts (NSCTs) became aware of, investigated and shared learning from deaths.[14] In response, the NHS launched a new programme of work to improve standards. This included national guidance on 'Learning from Deaths' (LfDs), providing a framework for NSCTs on 'identifying, reporting, investigating and learning from deaths in care'. The objectives of the guidance included supporting the NHS in England to develop an understanding of why deaths arising from problems in care occur, with the aim of ensuring that findings are shared and acted upon, to prevent recurrence.[15] In July 2017 guidance was published on implementing the LfDs framework at NSCT board level,[16] and amendments to statutory regulations followed. These changes made annual reporting of both quantitative and qualitative information relating to patient deaths a legal requirement in England (NHS quality account regulations 2010 (2017 No.744)).[17] The reporting mechanism was built into the NHS "Quality Accounts" system – where NSCTs are legally required to produce a publicly available annual report about the quality of their services (United Kingdom government legislation).[18]

Guidance was not given on expected number of deaths, how to judge if a death was more likely than not due to problems in care, or on examples of learning, actions or how to assess impact of any actions. It was instead left to individual NSCTs to decide how they would undertake these requirements. Guidance was given that NSCT board leadership should 'shares relevant learning across the organisation and with other services',[15] and that NSCTs should 'engage meaningfully with bereaved families and carers'.[19] It was not a statutory requirement to report on bereaved family and carer engagement or to report sharing of learning. This study analyses if NSCTs are reporting as legally required, evaluates the

For peer review only - http://bmjopen.bmj.com/site/about/guidelines.xhtml

quality of reporting, and determines whether there is evidence of effective organisational learning, sharing of learning and engagement with bereaved families and carers.

For peer review only

METHODS

This is a qualitative and quantitative study of an NHS safety improvement programme. We undertook analysis of 2017/2018 Quality Account data from NSCTs in England. We excluded Quality Accounts from ambulance trusts because in 2017/18 they were not required to report. This study has been reported using Standards for Reporting Qualitative Research.[20]

Our objectives were to describe the quality of reporting, and to thematically analyse the reports to derive key learning for the NHS and beyond. We undertook analysis of LfDs as set out in the 2017 amendment to the NHS 2010 Quality Account regulations.

Our evaluation of the quality of reporting involved review of compliance of reports against regulation numbers 27.1 to 27.6 (table 1).[17] Where NSCTs did not fully report we sought to understand why this may have been the case from comments within the Quality Account itself.

Table 1: NHS Quality Accounts LfDs Regulations [17]

Regulation number	Summary of regulatory requirement
**27.1**	The number of patients who have died during the annual reporting period
**27.2**	The number of the deaths (in 27.1) that have undergone a case record review or investigation
**27.3**	An estimate of the number of deaths in 27.2 which the NSCT judges to be more likely than not to have been due to problems in care, with explanation of method to assess this
**27.4**	What the NSCT has learnt from reviews/investigations in relation to deaths (in 27.3)
**27.5**	A description of the actions the NSCT has taken or will take in response to what they have learnt
**27.6**	An assessment of the impact of the actions (from 27.5)

In addition to statutorily required reporting we also looked for evidence within the 2017/18 LfDs report of family/carer engagement, which included evidence of involvement in learning and/or addressing family/carer concerns and/or appointing family liaison officer or similar as a result of a patient death. We also looked for evidence of sharing LfDs incidents both within the NSCT and more widely (for example with other organisations). Both sharing learning and family/carer engagement were recommended in the LfDs national guidance.[15]

Quantitative analysis of regulation 27.1- 27.3 was undertaken and reported using descriptive statistics.

Qualitative analysis to derive key learning themes from regulations 27.4 - 27.6, sharing learning and family/carer engagement was undertaken through document analysis as described by Bowen (2009) using both content and thematic analysis, and through exploratory data analysis. [21, 22] Both deductive and inductive approaches were utilised.

For peer review only

We first identified initial LfDs learning and action themes for reporting, and then developed a classification system for these. The first investigator (ZB) reviewed and analysed twenty 2017/2018 quality accounts, undertook open coding (inductive) and combined this with information presented at the NHS Improvement London LfDs Network (October 2018), where themes (mixed learning and action) from London NSCTs were discussed (deductive). Following the initial review, we reviewed the further 202 NSCT 2017/18 Quality Accounts. Each Account was reviewed by the same reviewer twice to ensure full data capture. Researchers used the process of bracketing to reduce subjective analysis.[23] During data capture further themes emerged, were modified, merged and changed iteratively. Recurring themes were identified using exploratory data analysis,[22] coding, identification of themes, recoding and using frequency charts. Data were captured in Microsoft excel (version 16.15).

Patient and public involvement

This study forms part of a larger programme of work which is overseen by a public and relatives steering group to improve relevance from the perspective of those affected by deaths in healthcare and to reduce biases from the healthcare staff researchers. The steering group have been involved in the planning, design and development of conclusions, through face-to-face meetings and email correspondence. The involvement of a steering group member in authoring this paper has significantly and positively influenced the reporting of this study, ensuring focus on reporting family involvement. The authors reflect that patient and public involvement (PPI) has been essential to this study to ensure that the views of bereaved family members were central to the concerns examined. The reporting of PPI has been undertaken using guidance for reporting involvement of patients and the public 2 – short form (GRIPP2-SF).[24]

For peer review only

RESULTS

Quality Accounts were reviewed for all 222 NSCTs in England.

Quality of Reporting

98 out of 222 (44%) NSCTs reported all six statutory elements of the LfD reporting framework. Two NSCTs did not report any parts of the LfDs regulatory requirements.[25, 26] The total number of deaths reported (regulation 27.1) varied from 3 deaths to 7756 deaths (median 1210.5, range 7753).[27, 28] The number of case record reviews or investigations undertaken relative to the number of patient deaths in individual NSCTs varied between 0.2% and 100% of deaths; the average was 43.7% (median 36.5, range 99.8).

Number of deaths which the NSCT judges to be more likely than not to have been due to problems in care, with explanation of method used to assess this

There was variation between 0 and 13% in the number of deaths which the NSCT judged to be more likely than not to have been due to problems in care (median 0.2, range 13). 22 NSCTs did not report any figure in this section of the quality accounts, reasons given for this included:

- 'data collection challenges'[29]
- 'unable to provide a reliable figure'[30]
- 'we do not carry out investigations with a view to determining whether the death was wholly or partly due to problems in the care provided'[31]
- 'currently no research base on this for mental health services and no consistent accepted basis for calculating this data'[32]

111 out of 222 NSCTs (50%) noted the use of Structured Judgement Reviews (SJRs) (either Royal College of Physicians or Royal College of Psychiatrists) either alone or in combination with other forms of investigation or review to assess problems in care.[33] NSCTs not using SJRs used a variety of other methods including: Confidential Enquiry into Stillbirths and Deaths in Infancy (CESDI) framework, Root Cause Analysis (RCA) and PReventable Incidents Survival and Mortality (PRISM) methodology.[34, 35]

Plans for assessment of impact

Regulation 26.6 asked NSCTs to undertake 'an assessment of the impact of the actions'. 105 out of 222 NSCTs (47%) discussed assessment of impact. This includes trusts that had a plan of any sort including a future plan. Several NSCTs used audits and/or quality improvement projects to check that actions are implemented. One NSCT stated 'Many of these actions are difficult to objectively assess in terms of their impact as they may relate to rare occurrences, which are difficult to meaningfully audit'.[36] The 47% of NSCTs who had a plan for assessment of impact does not include NSCTs that acknowledge the need to assess the impact but stated that it was too early to be able to undertake this (or words to this effect).[37, 38] Some NSCTs have reported the results of the assessment of impact that they have already undertaken.[39] Several NSCTs appear to have misunderstood, for example reiterating the purpose of the LfDs programme, instead of assessing impact.[40, 41]

For peer review only

Evidence involvement of family/carers in learning

In the 2017/18 LfDs reports 37 out of 222 NSCTs (17%) mentioned the involvement of families/carers either in the investigation process or in shared learning or that they communicate with/support/engage/consider families/carers after a patient dies.[42-44] A good example of working with families from one NSCT LfDs report states: 'The Trust continues to learn the importance of communication with families after a death has occurred and that through meaningful engagement after a death by inviting them to contribute to the terms of reference for investigations a more detailed, meaningful and richer account of the person's care and treatment is realised'.[45] One NSCT LfDs report discusses that as an action undertaken they sought to gain better education and training for staff about the importance of positive family engagement through expert external training.[46] 38 NSCTs (17%) discussed as an 'action' that they plan to work with/communicate with/engage/support families/carers. Many of these NSCTs are the same NSCTs already undertaking family/carer engagement.

Evidence learning shared more widely

In the 2017/18 trust LfDs reports 106 out of 222 NSCTs (48%) have shared or plan to share the learning more widely within their own organisation, through a variety of communication mediums: Face to face meetings or events, intranet (as case studies, safety alerts, newsletters).[36, 44, 47] 17 out of 222 (8%) NSCTs have shared or plan to share the learning outside their organisation, with neighbouring NSCTs or other national organisations.[47-50]

Key Findings from the Reports

Lessons learnt

Regulation 27.4 asks NSCTs to describe 'what the provider has learnt from reviews/investigations in relation to deaths' where this was related to deaths which the NSCT judged to be more likely than not to have been due to problems in care (regulation 27.3). 25 out of 222 NSCTs (11%) did not report any lessons learnt from deaths; of these 25 NSCTs, 9 NSCTs had reported 1 or more death judged to be more likely than not due to problems in care, the other 16 NSCTs had either reported zero deaths judged to be more likely than not due to problems in care or had not reported. However, 49 out of 222 NSCTs (22%) which reported that they had no deaths judged more likely than not due to problems in care, also reported lessons learnt, many caveating this with an explanation that they had learnt valuable lessons through the process of case note review/investigation. The most common learning themes from all NSCTs who reported learning can be found in table 2. An overview of the themes arising can be found in the frequency table (figure 1).

Table 2. The five most common learning themes across all NSCTs

Learning Themes	Number of NSCTs citing theme

Poor communication (including language barrier and problems with handover)	90 (46%)
Problem in recognition and escalation of deteriorating patients	83 (42%)
End of life planning or treatment escalation planning not evident/incomplete	82 (42%)
Problems with documentation including consent, details patient team and NOK	80 (41%)
Lack of clinical knowledge, consideration differential/delay diagnosis or seeking advise	53 (27%)

Some NSCTs have undertaken analysis of their learning and described common themes.[51] Some have gone into great detail.[52] Others have described a specific case or cases.[53] Some NSCTs have identified learning and actions together, without differentiating the learning from the action. The lack of structure in reporting makes it difficult to always understand exactly what the problem was leading to the learning.[54] Some NSCTs identified 'Good practice' as learning points.[55] Occasionally NSCTs did not necessarily learn from patient deaths, but from the overall LfDs process.[56]

Actions taken or planned to be taken

NSCTs were asked to undertake 'a description of the actions the NSCT has taken or will take in response to what they have learnt'. 30 out of the 222 NSCTs (14%) did not report any actions taken as a result of learning. One reported that they felt they were 'at too early a stage of development to be able to take actions from specific learning'.[37] The most common action themes from all NSCTs who reported actions can be found in table 3. An overview of the themes arising can be found in the frequency table (figure 2).

Table 3. The five most common action themes across all NSCTs

Action Themes	Number of NSCTs citing theme
Review of process/Standard Operating Procedure (SOP)/pathway	128 (67%)
Highlight guidelines or protocols/policy use of guideline/policies or protocols/treatment bundle/toolkit	96 (50%)
Implementation programme of work/education/bundle	96 (50%)
Quality improvement work or similar	90 (47%)
Work to improve communication/collaboration/shared learning	62 (32%)

The level of detail with regards to actions taken varies greatly with some NSCTs listing some specific actions as bullet points.[39] Others have described a specific case or cases.[57, 58]

For peer review only

DISCUSSION

This study demonstrates wide variation in both the quality of reporting and the findings from LfDs reports. Considering this is a new programme, introduced part-way through 2017/18, with limited guidance, the overall findings are somewhat encouraging. Nearly all NSCTs reported at least one or more element of the statutory LfD requirements. Most NSCTs reported lessons learnt and/or actions taken, while less than half discussed assessment of impact. The lessons learnt were varied. The most common learning theme reported was poor communication, with the most common action theme reported being; review of process/Standard Operating Procedure (SOP)/pathway.

Quality of Reporting

Reporting variation may be due to differences in interpretation of the guidance and statutory requirements. There is no direct financial penalty for a NSCT not reporting some or all elements of the LfDs statutory requirements in their Quality Accounts. However penalties can arise during CQC inspections, when an assessment of implementation of LfDs is carried out.[59, 60]

The different approaches taken by NSCTs and the heterogeneity of data makes comparison difficult. The variation in the percentage of deaths being reviewed/investigated may be due to some NSCTs not having the capacity to review/investigate cases, collect and/or report accurately. NSCTs with a very small number of deaths may find it easier to review all deaths than very large NSCTs. Some NSCTs have had mortality review processes in place for several years and have already been reviewing/investigating deaths, making implementation of the LfDs process easier since the structure for reviewing cases and personnel required are already in place. Some NSCTs may have felt at risk from negative attention by declaring total numbers of deaths and deaths judged more likely than not due to problems in care. Many NSCTs did however report despite the same risk. It is clear from the LfDs reports that several NSCTs, particularly some mental health and community NSCTs, did not feel that the guidance applied to them, however other similar NSCTs were able to comply with reporting. The results could suggest guidance was written with acute NSCTs in mind and perhaps need to be reconsidered for non-acute NSCTs. Similar findings were noted by the CQC in their report 'Learning from deaths: A review of the first year of NHS trusts implementing the national guidance'.[60]

The variation in deaths judged more likely than not due to problems in care is larger than those noted in previous studies.[2-6] It seems unlikely than many NSCTs would experience no deaths judged more likely than not due to problems in care. This could realistically be the case in specialist NSCTs where the absolute number of total deaths is very small, or community NSCTs with no inpatient beds, but seems unlikely in large acute NSCTs. Despite the improbability several acute NSCTs did report zero deaths judged more likely than not due to problems in care. Further work to understand why these NSCTs reported zero deaths should be undertaken.

The element of the statutory LfDs reporting that prompted poor responses from most NSCTs was 'An assessment of the impact of the actions' and describing how they would undertake this. The vast majority of NSCTs have answered this in a vague manner, seemingly through variable interpretation of the regulation. Improvements could be made by issuing further

For peer review only

specific guidance in relation to this element of the reporting. Of the NSCTs who did manage to implement actions and assess impact this was often using quality improvement measurements. The use of quality improvement methodology is felt to be an important overall indicator of quality by the CQC.[61] Guidance on evaluating the impact of interventions is widely available.[62, 63]

Collectively within the LfDs reports, there is much learning, some resulting in impactful actions and high-level organisational learning.[8] This learning could potentially be usefully shared across the NHS and internationally. Some NHS NSCTs appear to have disengaged with the programme. This study suggests a lack of shared learning from the LfDs reports particularly between NSCTs and a lack of family engagement, despite NHS guidance.[19] Since the involvement of families and sharing learning were not statutory requirements of LfDs reporting, they may be underrepresented in the LfDs reports, this should be investigated further before any definite conclusions can be drawn about NSCTs engagement in this element of the LfDs guidance. This study does demonstrate an apparent disparity in organisational learning and safety culture, which results in inequity for families/carers. This should be addressed by the DHSC and associated national bodies. Since the oversight bodies which were established to support the programme in its initial stages have now been stood down this seems unlikely to happen.[64, 65]

Key Findings from the Reports

Overall consistency with regards to identifying, reporting, investigating, learning from deaths in care and taking action has improved across most NSCTs. The continual process of learning, action and reflection which characterises effective organisational learning is essential to ensure the change necessary for safer healthcare. This can only be achieved where information and knowledge affecting patient safety is easily accessible to all members of healthcare staff, supporting an overall safety culture.[66, 67]

Only a small number of NSCTs did not report any learning, suggesting that most NSCTs were able to engage with this aspect of reporting. Many NSCTs have effectively described lessons learnt and actions taken. However, most of the LfDs report recommendations or actions are fairly non-specific; further detail of actions and their measurable impact would be helpful.

It is of concern that the majority of these lessons and recommended actions have previously been identified in national and international reports and inquiries, looking at the problems associated with preventable deaths. Similar problems found in this study are also highlighted in these reports; poor clinical monitoring, poor recognition of the deteriorating patient, diagnostic errors, poor communication, lack of end of life planning, lack of information sharing between services, inadequate drug and fluid management.[68-76] This suggests many of the same problems reoccur and that healthcare systems do not learn from previous failings and adds weight to the proposition that the NHS as a whole cannot become a learning organisation.[77] In view of this, it is reasonable to question whether the learning arising from LfDs reporting will result in meaningful change. If LfDs findings and recommendations are not implemented, systemic redundancy in the initiative is implied. While individual healthcare practitioners do need to take some responsibility, NSCTs and the DHSC should look at systems, such as institutional accountability and LfDs programme oversight to optimise outcomes and minimise the risk of fatal patient safety incidents occurring. This lack of change adds to the growing body of evidence suggesting that traditional approaches to

For peer review only

organisational learning in healthcare, such as learning from when things go wrong (safety-I) have limited effect and may suggest a role for increased learning from the patients who have experienced excellent patient care and outcome despite being seriously unwell (safety-II).[78]

Recommendations

In view of the findings from this study, in order to improve reporting quality, our recommendations are as follows:

- A more structured LfDs reporting template, including all regulatory requirements should be implemented through the Quality Accounts
- NHSE/I specific guidance should be developed on how NSCTs can undertake 'an assessment of the impact of the actions'
- To reinstate LfDs robust regulatory reporting oversight in addition to CQC inspections

In order to improve 'learning and action' from deaths, our recommendations are:

- Annual collection and collation of all NSCT LfDs reporting that is made publicly available
- Further investigation into how NSCTs currently involve bereaved families and carers
- Investment in leadership and support for NHS staff to enable a safety culture

Methodological Limitations

This is an analysis of the very first year of LfDs reporting and reports could underrepresent current NSCT engagement in the LfDs process. NSCTs may be undertaking elements of the LfDs programme that were not statutory reporting requirements such as family/carer engagement, but not reporting on these as it was not a regulatory requirement. NSCT LfDs reports were not created for research analysis and are not standardised, this heterogeneity and subjectivity within the reports reduces equitable comparison.

Despite attempts to minimise inherent researcher bias, such as through PPI involvement and the process of bracketing, the qualitative analysis may have been influenced to a limited extent.

For peer review only

CONCLUSION

Organisations are variably reporting against LfDs regulations, with overall improved consistency in the way that NSCTs identify, report, investigate and learn from deaths in care since the CQC review. [14] However, more could be done to enhance and strengthen the programme impact, and to assess whether LfDs reporting reflects NSCT LfDs engagement.

On the basis of findings from the 2017/18 LfDs reports, national programmes led by multidisciplinary healthcare practitioners should be developed to tackle the most common problems which may have contributed to patient deaths. In the first instance programmes tackling the following issues should be developed or strengthened:

- Improving communication [79]
- Involvement of families in care and in learning [80]
- Processes to share learning (locally and nationally) [81]

Further work is needed to understand which actions taken by NSCTs result in the biggest impact and for this learning to be shared. While LfDs can be difficult and emotive it is fundamental that healthcare systems ensure learning and impactful change occur.

ACKNOWLEDGEMENTS

We would like to acknowledge the work of the Learning from Deaths: Learning and Action (LfDLaA) Public and Relatives steering group in this research. Patient and public involvement in this research was supported by the NIHR UCL Biomedical Research Centre.

For peer review only

REFERENCES

1 World Health Organisation 2019. Patient safety. Accessed July 2020. Available at: https://www.who.int/news-room/fact-sheets/detail/patient-safety
2 Levinson DR 2010. Adverse events in hospitals: national incidence among Medicare beneficiaries. Office of Inspector General for the Department of Health and Human Services. Accessed July 2020. Available at: https://oig.hhs.gov/oei/reports/oei-06-09-00090.pdf
3 Kobewka DM, Van Walraven C et al. The prevelance of potentially preventable deaths in an acute care hospital.: A retrospective cohort. Medicine. 2017 Feb;96(8):e6162. doi:1097/MD.0000000000006162
4 Hogan H, Healey F et al. Preventable deaths due to problems in care in English acute hospitals: retrospective case record review study. BMJ Quality & Safety 2012;21:737-745. doi:10.1136/bmjqs-2011-001159
5 Roberts AP, Morrow G et al. From research to practice: results of 7300 mortality retrospective case record reviews in four acute hospitals in the North-East of England. BMJ Open Qual.2017;6(2):e000123. doi:10.1136/bmjoq-2017-000123
6 Rogne T, Nordseth T et al. Rate of avoidable deaths in a Norwegian hospital trust as judged by retrospective chart review. BMJ Quality & safety. 2019 Jan;28(1):49-55. doi:10.1136/bmjqs-2018-008053
7 NHS Digital 2018. Summary Hospital-level Mortality Indicator (SHMI) - Deaths associated with hospitalisation, England, April 2017 - March 2018. Accessed July 2020. Available at: https://digital.nhs.uk/data-and-information/publications/clinical-indicators/shmi/archive/shmi-april-2017---march-2018
8 Fiol M, Lyles MA. Organizational Learning. The Academy of Management Review. Oct 1985;10(4):803-813
9 Salancik GR, Mendl JR. Corporate attributions as strategic illusions of management control. Administrative Science Quarterly 1984;29:238-254
10 Carroll JS, Edmondson AC. Leading organisational learning in health care. Qual Saf Health Care 2002;11:51–56. doi:10.1136/qhc.11.1.51
11 Mannion R, Braithwaite J. Where is Patient Safety Research and Practice Heading? A Response to Recent Commentaries. Int J Health Policy Manag 2019;8(2):136-137. doi: 10.15171/ijhpm.2018.112
12 Hamblin R, Shuker C Well spotted: but now you need to do something BMJ Quality & Safety Published Online First: 18 September 2020. doi: 10.1136/bmjqs-2019-010659
13 Mazars 2015. Independent review of deaths of people with a Learning Disability or Mental Health problem in contact with Southern Health NHS Foundation Trust April 2011 to March 2015. Accessed July 2020. Available at: https://www.england.nhs.uk/south/wp-content/uploads/sites/6/2015/12/mazars-rep.pdf
14 CQC 2016. Learning, candour and accountability. Accessed July 2020. Available at: https://www.cqc.org.uk/sites/default/files/20161213-learning-candour-accountability-full-report.pdf
15 NQB 2017. National Guidance on Learning from Deaths. Accessed July 2020. Available at: Accessed July 2020. Available at: https://www.england.nhs.uk/wp-content/uploads/2017/03/nqb-national-guidance-learning-from-deaths.pdf
16 NHS Improvement 2017. Implementing the Learning from Deaths framework: key requirements for trust boards. Accessed July 2020. Available at: https://improvement.nhs.uk/documents/1747/170921__Implementing_LfD_-_information_for_boards_JH_amend_3.pdf

For peer review only

17 UK Legislation 2017. The National Health Service (Quality Accounts) (Amendment) Regulations 2017. Accessed July 2020. Available at: http://www.legislation.gov.uk/uksi/2017/744/made
18 NHS 2019. About Quality Accounts. Accessed July 2020. Available at: https://www.nhs.uk/using-the-nhs/about-the-nhs/quality-accounts/about-quality-accounts/
19 NQB 2018. Learning from deaths: Guidance for NHS trusts on working with bereaved families and carers. Accessed Sept 2020. Available at: https://www.england.nhs.uk/wp-content/uploads/2018/08/learning-from-deaths-working-with-families-v2.pdf
20 O'Brien BC, Harris IB et al. Standards for reporting qualitative research: a synthesis of recommendations. Acad Med. 2014;89(9):1245-1251. doi:10.1097/ACM.0000000000000388
21 Bowen GA. 2009. Document Analysis as a Qualitative Research Method, Qualitative Research Journal. 9 (2). 27-40. DOI:10.3316/QRJ0902027
22 Stebbins R. Exploratory Data Analysis. In: Given LM, The Sage Encyclopedia of Qualitative Research Methods, Vol. 1, Ed. 1. Sage 2008:325-326
23 Tufford L, Newman P. Bracketing in Qualitative Research. Qualitative Social Work 2010;11:80-96. doi:10.1177/1473325010368316
24 Staniszewska S, Simera I et al. GRIPP2 reporting checklists: tools to improve reporting of patient and public involvement in research. BMJ 2017;358:j3453. doi: 10.1136/bmj.j3453
25 Worcestershire Acute Hospitals NHS Trust Quality Account 2017/18. Accessed July 2020. Available at: https://www.worcsacute.nhs.uk/stoma-care/2146-quality-account-2017-18/file
26 North Staffordshire Combined Healthcare NHS Trust Quality Account 2017/18. Accessed July 2020. Available at: http://389w1t43oria4ctlpf36b5lz-wpengine.netdna-ssl.com/wp-content/uploads/2018/10/Combined-Quality-Account-2017-18.pdf
27 Moorfields Eye Hospital NHS Foundation Trust Quality Account 2017/18. Accessed July 2020. Available at: https://www.moorfields.nhs.uk/sites/default/files/Moorfields%20annual%20report%202017-18.pdf
28 North East London Foundation Trust Quality Account 2017/18. Accessed July 2020. Available at: https://www.nelft.nhs.uk/download.cfm?doc=docm93jijm4n3126.pdf&ver=5090
29 North Middlesex University Hospital NHS Trust Quality Account 2017/18. Accessed July 2020. Available at: http://www.northmid.nhs.uk/Portals/0/201809%20-%20NMUH%20Final%20Quality%20Account%202017-18.pdf?ver=2018-09-18-125322-917
30 Royal Devon and Exeter NHS Foundation Trust Quality Account 2017/18. Accessed July 2020. Available at: https://www.rdehospital.nhs.uk/documents/trust/documents/annual-report-2017-2018.pdf
31 Black Country Partnership NHS Foundation Trust Quality Account 2017/18. Accessed July 2020. Available at: https://www.bcpft.nhs.uk/documents/about-us/key-documents/annual-report/1851-bcp-annual-report-2018/file
32 Sheffield Health and Social Care NHS Foundation Trust Quality Account 2017/18. Accessed July 2020. Available at: https://www.shsc.nhs.uk/sites/default/files/2019-12/Quality%20Report%202017-2018.pdf
33 Healthcare Quality Improvement Partnership 2018. Royal College of Physicians: National mortality case record review. Annual report. Accessed July 2020. Available at: https://www.rcplondon.ac.uk/projects/outputs/national-mortality-case-record-review-nmcrr-annual-report-2018
34 MBRRACE-UK 2017. Perinatal Confidential Enquiry. Confidential Enquiry into Stillbirths and Deaths in Infancy (CESDI) framework:19-20. Accessed July 2020. Available

For peer review only

at: https://www.npeu.ox.ac.uk/downloads/files/mbrrace-uk/reports/MBRRACE-UK%20Intrapartum%20Confidential%20Enquiry%20Report%202017%20-%20final%20version.pdf
35 Hogan H 2014. Preventable, Incidents, Survival and Mortality Study 2 (PRISM) Medical Record Review Manual. Accessed July 2020. Available at: https://improvement.nhs.uk/documents/1423/PRISM_2_Manual_V2_Jan_14.pdf
36 University Hospital Southampton NHS Foundation Trust Quality Account 2017/18. Accessed July 2020. Available at: https://www.uhs.nhs.uk/Media/SUHTInternet/AboutUs/AnnualReportsStrategiesandPlans/201718/Annual-report-2017-18.pdf
37 Royal United Hospitals Bath NHS Foundation Trust. Accessed July 2020. Available at: https://www.ruh.nhs.uk/about/annual_report/documents/Annual_Report_2017-18.pdf
38 Great Western Hospitals NHS Foundation Trust Quality Account 2017/18. Accessed July 2020. Available at: https://www.gwh.nhs.uk/media/309191/gwhnhsftannualqualityaccounts2017-18.pdf
39 Salisbury NHS Foundation Trust Quality Account 2017/18. Accessed July 2020. Available at: https://www.salisbury.nhs.uk/AboutUs/TrustReportsAndReviews/Documents/SalisburyNHSFoundationTrustQualityAccount2017_2018.pdf
40 Gloucestershire Hospitals NHS Foundation Trust Quality Account 2017/18. Accessed July 2020. Available at: https://www.gloshospitals.nhs.uk/media/documents/QA-A4-17-18.pdf
41 Isle of Wight NHS Trust Quality Account 2017/18. Accessed July 2020. Available at: https://www.iow.nhs.uk/Downloads/Quality%20Account/Quality%20Accout%202017-18.pdf
42 Tameside and Glossop Integrated Care NHS Foundation Trust Quality Account 2017/18. Accessed July 2020. Available at: https://www.tamesidehospital.nhs.uk/Documents/AnnualReport20172018.pdf
43 Buckinghamshire Healthcare NHS Trust Quality Account 2017/18. Accessed July 2020. Available at: https://www.buckshealthcare.nhs.uk/Downloads/quality-account/08%20Quality%20Accounts_2017_18.pdf
44 Birmingham Women's and Children's NHS Foundation Trust Quality Account 2017/18. Accessed July 2020. Available at: https://bwc.nhs.uk/download.cfm?doc=docm93jijm4n2490.pdf&ver=3456
45 Pennine Care NHS Foundation Trust Quality Account 2017/18. Accessed July 2020. Available at: https://www.penninecare.nhs.uk/application/files/4615/6172/0636/Quality_account_2017-18.pdf
46 South Staffordshire and Shropshire Healthcare NHS Foundation Trust Quality Account 2017/18. Accessed July 2020. Available at: https://improvement.nhs.uk/documents/4294/South_Staffordshire_Shropshire_Healthcare_Annual_Report_and_Accounts_2017-18.PDF
47 Royal Free London NHS Foundation Trust Quality Account 2017/18. Accessed July 2020. Available at: https://www.royalfree.nhs.uk/uploads/RF_quality_report_2017-18.pdf
48 South west Yorkshire Partnership NHS Foundation Trust Quality Account 2017/18. Accessed July 2020. Available at: https://www.southwestyorkshire.nhs.uk/wp-content/uploads/2018/08/SWYPT-annual-report-and-accounts-2017-18-FINAL.pdf
49 Northumbria Healthcare NHS Foundation Trust Quality Account 2017/18. Accessed July 2020. Available at: https://www.northumbria.nhs.uk/wp-content/uploads/2018/07/FINAL-NHFT-ARA-2017-18.pdf

For peer review only

50 Greater Manchester Mental Health NHS Foundation Trust Quality Account 2017/18. Accessed July 2020. Available at: https://www.gmmh.nhs.uk/download.cfm?doc=docm93jijm4n3649.pdf&ver=7138
51 The Christie NHS Foundation Trust Quality Account 2017/18. Accessed July 2020. Available at: https://www.christie.nhs.uk/media/5682/the-christie-nhs-ft-quality-report-2017-18.pdf
52 Northumberland, Tyne & Wear NHS Foundation Trust Quality Account 2017/18. Accessed July 2020. Available at: https://improvement.nhs.uk/documents/4419/Northumberland_Tyne___Wear_NHS_FT_Annual_Report_and_Accounts_2017-18.PDF
53 The Walton Centre NHS Foundation Trust Quality Account 2017/18. Accessed July 2020. Available at: https://www.thewaltoncentre.nhs.uk/uploadedfiles/THE%20WALTON%20CENTRE%20FINAL%20MERGED%20ANNUAL%20REPORT%20ACCOUNTS%20QUALITY%20ACCOUNT%202017_18.pdf
54 Kent & Medway NHS and Social Care Partnership Trust Quality Account 2017/18. Accessed July 2020. Available at: https://www.kmpt.nhs.uk/media/1234/quality-account-17-18.pdf
55 Bradford Teaching Hospitals NHS Foundation Trust Quality Account 2017/18. Accessed July 2020. Available at: https://www.bradfordhospitals.nhs.uk/wp-content/uploads/2018/07/Annual-Report-and-Accounts-2017-18.pdf
56 Maidstone and Tunbridge Wells NHS Trust Quality Accounts 2017/18. Accessed July 2020. Available at: https://www.mtw.nhs.uk/wp-content/uploads/2018/06/MTW-Quality-Accounts-2018.pdf
57 Cambridge University Hospitals NHS Foundation Trust Quality Account 2017/18. Accessed July 2020. Available at: https://www.cuh.nhs.uk/sites/default/files/Annual%20Report%20and%20Accounts%20201718.pdf
58 Camden and Islington NHS Foundation Trust Quality Account 2017/18. Accessed July 2020. Available at: https://www.cuh.nhs.uk/sites/default/files/Annual%20Report%20and%20Accounts%20201718.pdf
59 CQC 2018. Inspection framework: NHS trusts and foundation trusts. Trust-wide well-led. Accessed July 2020. Available at: https://www.cqc.org.uk/sites/default/files/20200115_Trust_wide_well_led_inspection_framework_V7.pdf
60 CQC 2019. Learning from deaths: A review of the first year of NHS trusts implementing the national guidance Accessed July 2020. Available at: https://www.cqc.org.uk/sites/default/files/20190315-LfD-Driving-Improvement-report-FINAL.pdf
61 CQC 2018. Quality improvement in hospital trusts. Accessed July 2020. Available at: https://www.cqc.org.uk/sites/default/files/20180911_QI_hospitals_FINAL.pdf
62 NHS 2015. How to understand and measure impact. Accessed Sept 2020. Available at: https://www.england.nhs.uk/wp-content/uploads/2015/06/bcf-user-guide-04.pdf.pdf
63 Clarke Geraldine M, Conti Stefano et al. Evaluating the impact of healthcare interventions using routine data BMJ 2019;365:l2239. doi: 10.1136/bmj.I2239
64 RCP 2019. National Mortality Case Record Review Programme. Accessed July 2020. Available at: https://www.rcplondon.ac.uk/projects/national-mortality-case-record-review-programme

For peer review only

65* Ocloo J, Smith D. Bereaved families have been used as pawns in a political game. Health Service Journal. February 2021. Accessed February 2021. Available at: https://www.hsj.co.uk/patient-safety/bereaved-families-have-been-used-as-pawns-in-a-political-game/7029561.article
66 Carroll JS, Edmondson AC. Leading organisational learning in healthcare. BMJ Quality & Safety 2002;11(1):51-6. doi: 10.1136/qhc.11.1.51
67 Mannion R, Konteh FH, Davies HTO. Assessing organisational culture for quality and safety improvement: a national survey of tools and tool use. BMJ Quality & Safety 2009;153:153-6. doi: 10.1136/qshc.2007.024075
68 National Confidential Enquiry into Patient Outcomes and Deaths (NCEPOD) 2018. Themes and recommendations common to all hospital specialties. Accessed July 2020. Available at: https://www.ncepod.org.uk/CommonThemes.pdf
69 NHS England 2014. Patient safety alert. Stage three: directive. Improving medication error incident reporting and learning. Accessed July 2020. Available at: https://www.england.nhs.uk/wp-content/uploads/2014/03/psa-sup-info-med-error.pdf
70 Singh H, Schiff GD, Graber ML, Onakpoya I, Thompson MJ. The global burden of diagnostic errors in primary care. BMJ Qual Saf 2017;26:484-494. doi: 10.1136/bmjqs-2016-005401
71 Oates A. Learning from suicide-related claims. A thematic review of NHS Resolution data. NHS Resolution. September 2018. Accessed July 2020. Available at: https://resolution.nhs.uk/wp-content/uploads/2018/09/NHS-Resolution_learing_from_suicide_claims_148pp_ONLINE1.pdf
72 Government of Western Australia, Department of Health 2019. From death we learn 2018. Accessed July 2020. Available at: https://ww2.health.wa.gov.au/~/media/Files/Corporate/Reports%20and%20publications/PDF/From-Death-We-Learn-2018.pdf
73 PHSO 2014. An avoidable death of a three-year-old child from sepsis. Accessed July 2020. Available at: https://www.ombudsman.org.uk/sites/default/files/An_avoidable_death_of_a_three_year_old_child_from_sepsis%20_report.pdf
74 Francis R. Report of the Mid Staffordshire NHS Foundation Trust Public Inquiry: Executive summary. Department of Health, 2013. Accessed July 2020. Available at: https://assets.publishing.service.gov.uk/government/uploads/system/uploads/attachment_data/file/279124/0947.pdf
75 Kirkup B. The Report of the Morecambe Bay Investigation. Department of Health, 2015. Accessed July 2020. Available at: https://assets.publishing.service.gov.uk/government/uploads/system/uploads/attachment_data/file/408480/47487_MBI_Accessible_v0.1.pdf
76 Walshe K, Higgins J. The use and impact of inquiries in the NHS. BMJ 2002;325(7369): 895–900. doi: 10.1136/bmj.325.7369.895
77 Sheaff R, Pilgrim D. Can learning organisations survive in the newer NHS? Implementation science 2006:1(27). doi: 10.1186/1748-5908-1-27
78 Sujan M. A Safety-II perspective on organisational learning in healthcare organisations. Int J Health Policy Manag 2018;7(7):662-666. doi: 10.15171/ijhpm.2018.16
79 NHS England 2020. The NHS Engagement and Communications Development Programme. Accessed February 2021. Available at: https://www.england.nhs.uk/nhs-communications-development/
80 Making Families Count. Accessed February 2021. Available at: https://www.makingfamiliescount.org.uk

For peer review only

81 NHS Education for Scotland. The Knowledge Network. Accessed February 2021. Available at: http://www.knowledge.scot.nhs.uk/adverse-events/sharing-learning.aspx

For peer review only

Footnotes

Contributors

ZB conceived and designed the study, undertook analysis, interpretation of the data and drafted the manuscript.
CVP provided critical input to the design, analysis, interpretation of the data and revised the manuscript critically.
DB provided critical input to the design, analysis, interpretation of the data and revised the manuscript critically.
SRM provided critical input to the design, analysis, interpretation of the data and revised the manuscript critically.
The corresponding author attests that all listed authors meet authorship criteria and that no others meeting the criteria have been omitted. ZB is the guarantor.

Funding

This study was supported by the National Institute of Health Research (NIHR) University College London (UCL) Biomedical Research Centre Patient and Public Involvement starter funding (BRC617/PPI/ZB/104990).

Competing interests

ZB worked at NHS Improvement in the medical directorate from August 2017 to August 2018, during which time she undertook some work on the LfDs programme. SRM is the National Clinical Director for Critical and Perioperative Care for NHS England/NHS Improvement, SRM has no link with the LfDs programme. CVP and DB do not have any competing interests to declare. All authors have completed the ICMJE uniform disclosure form at www.icmje.org/coi_disclosure.pdf

Ethical approval

This study is an analysis of publicly available data and does not require ethical approval.

Data availability statement

All the data for this study is publicly available from the 2017/18 NHS Quality Accounts.

For peer review only

Figure Legends

Figure 1. Frequency table of lessons learnt (all NSCTs n=222)

A – Problem in recognition & escalation of deteriorating patients
B – Lack of or awareness of or following protocol/guideline/bundle
C – Problem in assessment or experience related to learning disabilities
D – Poor communication (including language barrier & problems with handover)
E – Problem with end-of-life planning or treatment escalation planning
F – Problem with death certification or confirming death
G – Problem with discharge (timing/letters/delay/information for patients)
H – Difficulty accessing support services/ Out of Hours services/ Specialist services
I – Problem/lack of risk assessment/interventions
J – Lack of knowledge of hospital layout/equipment
K – Problem with patient transfers
L – Problem assessing/providing nutrition/fluids/electrolytes
M – Lack of senior/consultant review, input, planning
N – Excellent/good care/management
O – Prompt senior review
P – Good communication/collaboration/teamwork
Q – Lack of clinical knowledge, consideration differential diagnosis or seeking advise
R – Problem with/lack of prescribing or side-effects or administration of medications
S – Problem with 'Duty of Candour'
T – Delay to acting on results
U – Problems with documentation including consent
V – Delay/problem in requesting or interpretation of investigations
W – Lack of/problem with monitoring/observations/recording
X - Lack of/or problem with sharing information with other providers/services/specialties
Y – Delay in reviewing patient
Z – Delay in treatment/incomplete management including care plans and pain management
AA – Poor continuity of care/team work
AB – Concerns with pre-hospital care (residential settings/wider societal issues)
AC – Lack of familiarity with or standardisation or availability of equipment
AD – Problem related to workforce or staffing or supervision of staff
AE – Misfiled documents/lost notes/problems in storage or access of notes/scans
AF – Problem with recognition/management of Acute Kidney Injury
AG – Lack of Multidisciplinary Team involvement/discussion/decision
AH – Problem with competency or complication in undertaking procedure/operation
AI – Problem related to infection control
AJ – Lack of/problem with assessment of mental health needs and/or follow-up
AK – Problem related to appropriateness of patient ward allocation or relocation
AL – Problem with pre-op assessment/peri-op management
AM – Problem with capacity/flow/hospital of department pressures (including A&E)
AN – Deviation from treatment plan or plan not linked with clinical record
AO - Follow-up planning not evident or incomplete/problem with follow-up
AP – Problem related to management of physical health problem in mental health setting
AQ – Problem due to patient not wanting to/unable to engage with treatment (with capacity)
AR – Problem after death (related to post-mortem/forensic services or investigation)
AS – Problem with the recognition/management of drug/alcohol withdrawal/recovery
AT – Lack of supervision or safe accommodation for vulnerable patient

For peer review only

AU – Lack of/problem with engagement with/support of families/carers

Figure 2. Frequency table of actions taken (all NSCTs n=222)

A – Work to improve communication/collaboration/shared learning
B – Improved end-of-life planning (including communication)
C – Improved effectiveness of handover
D– Highlight or new or use of guidelines/protocols/policy/protocol/treatment bundle/toolkits
E – Improved mortality review process
F – Undertake or improve risk assessment/governance process/reporting system
G – Review of process/SOP/pathway/audit process
H – Quality improvement work or similar
I – 'Raising awareness' or 'Importance of' or 'reflecting on' (not qualified)
J – Implementation of a programme of work or education (including simulation & induction)
K – Raising awareness (with specific example – 'nursing dashboard', 'case presentation')
L – Use of technology (for example electronic recording of observations)
M – Rota adjusted to provide better cover or extra lists/sessions
N – Working/communicating with/supporting families (not end-of-life planning)
O – 'More effective', 'continued efforts', 'seeking advice' 'review/introduce' (not qualified)
P – Solution involving medical examiner role
Q – Improved senior/consultant involvement (with specific examples)
R – External or internal (peer review) mortality/governance review or investigation
S – Identification of high-risk patients early
T – Extend post-op recovery monitoring
U – Improved documentation/coding
V – Follow-up of action plans
W – Plan to improve sharing of learning
X – Ensure Early Warning System in place/utilised correctly
Y – Improvement of results reporting & acknowledgement process/archiving results/scans
Z – Multidisciplinary team/programme of work set-up to address specific problem
AA – Seek-out and follow expert advise
AB – Improve review methodology (Such as Structured Judgement Review training)
AC – Develop regional Learning from Deaths network or similar
AD – Negotiate with coroner for earlier post-mortem reports
AE – Increase emergency operating capability (additional emergency theatre availability)
AF – Improvement to bereavement facilities
AG – Improved infection control measures
AH – Supervision discussions/support/feedback for those involved in incidents
AI – Improved cross-specialty collaboration
AJ – Increased specialist equipment availability or specialist teams or specialist roles
AK – Increased engagement in LeDeR process

For peer review only

Figure 1. Frequency table of lessons learnt (all NSCTs n=222)

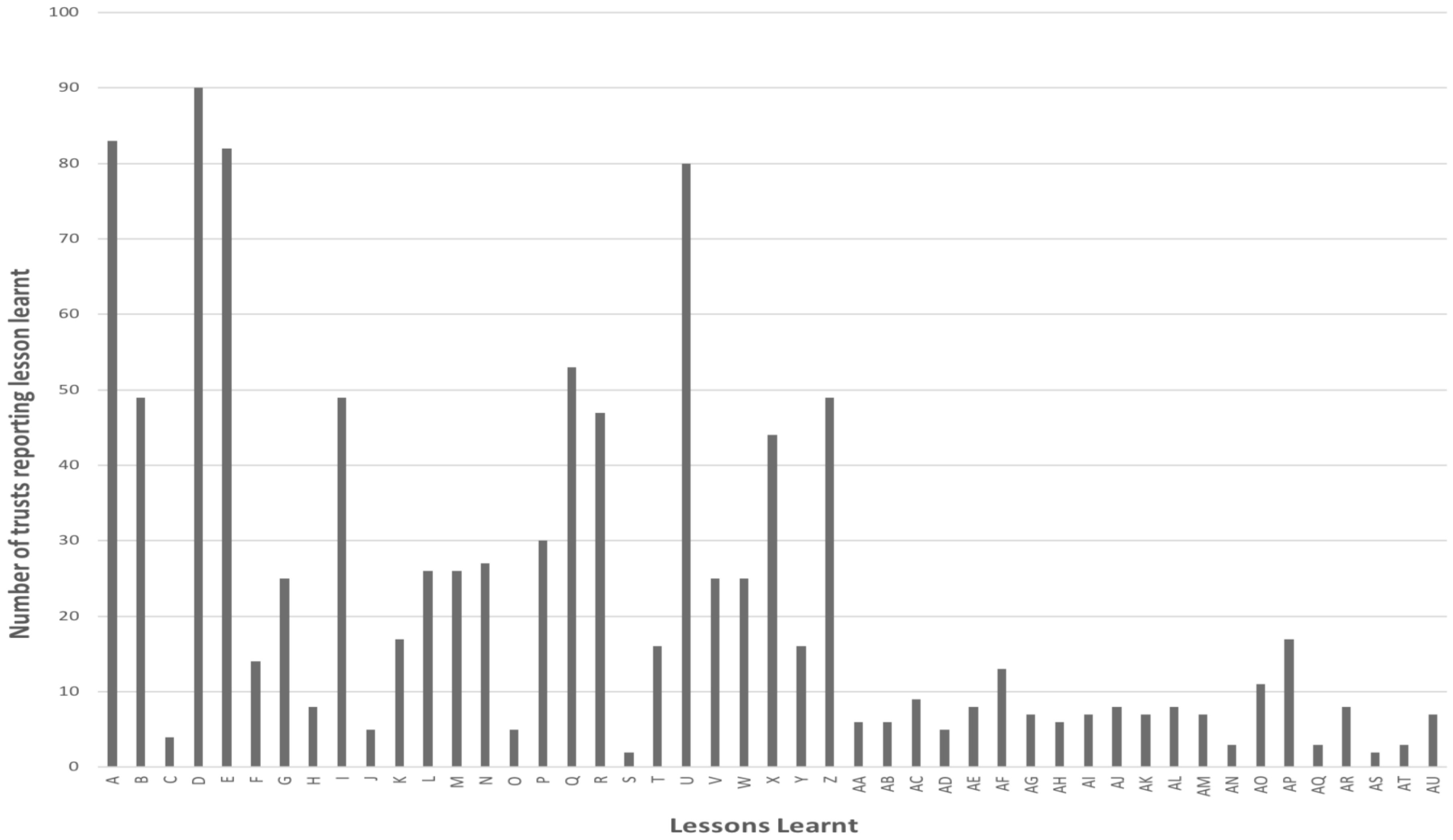

Figure 2. Frequency table of actions taken (all NSCTs n=222)

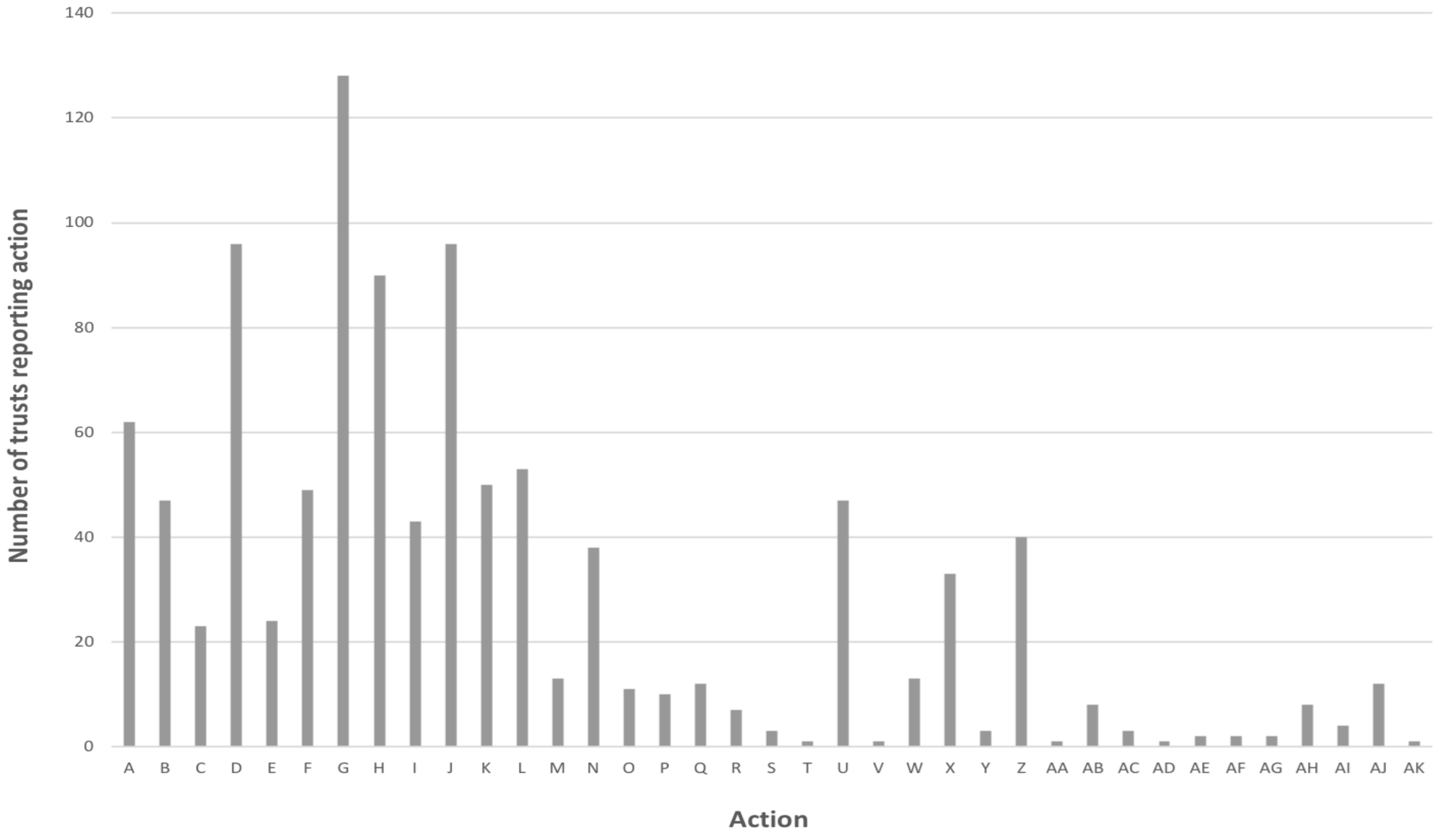

Standards for Reporting Qualitative Research (SRQR)

O'Brien BC, Harris IB et al. Standards for reporting qualitative research: a synthesis of recommendations. Acad Med. 2014;89(9):1245-1251. doi:10.1097/ACM.0000000000000388

**No.**	**Topic**	**Item**	**Page number and line in 030321.BMJOpen_LfDs where relevant information can be found**
**Title and abstract**			
**S1**	Title	Concise description of the nature and topic of the study Identifying the study as qualitative or indicating the approach (e.g., ethnography, grounded theory) or data collection methods (e.g., interview, focus group) is recommended	Page 1, lines 1-3
**S2**	Abstract	Summary of key elements of the study using the abstract format of the intended publication; typically includes background, purpose, methods, results, and conclusions	Page 2
**Introduction**			
**S3**	Problem formulation	Description and significance of the problem/phenomenon studied; review of relevant theory and empirical work; problem statement	Page 4, lines 3 - 10 and lines 23 - 40
**S4**	Purpose or research question	Purpose of the study and specific objectives or questions	Page 4, lines 49 and 50<br>Page 5, lines 1 and 2
**Methods**			
**S5**	Qualitative approach and research paradigm	Qualitative approach (e.g., ethnography, grounded theory, case study, phenomenology, narrative research) and guiding theory if appropriate; identifying the research paradigm (e.g., postpositivist, constructivist/interpretivist) is also recommended; rationale	Qualitative approach described:<br>Page 6, lines 32 - 35<br>Page 7, lines 1 - 11<br><br>Research paradigm not appropriate in this study.
**S6**	Researcher characteristics and reflexivity	Researchers' characteristics that may influence the research, including personal attributes, qualifications/experience, relationship with participants, assumptions, and/or presuppositions; potential or	Page 7, line 8<br>Page 21, lines 24 - 29

For peer review only

		actual interaction between researchers' characteristics and the research questions, approach, methods, results, and/or transferability	
**S7**	Context	Setting/site and salient contextual factors; rationale	Page 6, lines 4 - 6
**S8**	Sampling strategy	How and why research participants, documents, or events were selected; criteria for deciding when no further sampling was necessary (e.g., sampling saturation); rationale	Page 6, lines 4 - 6
**S9**	Ethical issues pertaining to human subjects	Documentation of approval by an appropriate ethics review board and participant consent, or explanation for lack thereof; other confidentiality and data security issues	Page 21, line 33
**S10**	Data collection methods	Types of data collected; details of data collection procedures including (as appropriate) start and stop dates of data collection and analysis, iterative process, triangulation of sources/methods, and modification of procedures in response to evolving study findings; rationale	Page 6, lines 4 – 7, lines 13 – 16, table 1, lines 22 – 27, lines 29 – 30, lines 32 – 35<br>Page 7, lines 1 - 11
**S11**	Data collection instruments and technologies	Description of instruments (e.g., interview guides, questionnaires) and devices (e.g., audio recorders) used for data collection; if/how the instrument(s) changed over the course of the study	Page 7, line 11
**S12**	Units of study	Number and relevant characteristics of participants, documents, or events included in the study; level of participation (could be reported in results)	Page 4, lines 37 - 40<br>Page 6, lines 4 – 6, lines 13 – 16, table 1, lines 22 - 27<br>Page 8, line 3, line 8
**S13**	Data processing	Methods for processing data prior to and during analysis, including transcription, data entry, data management and security, verification of data integrity, data coding, and anonymization/deidentification of excerpts	Page 6, lines 29 – 30, lines 32 – 35<br>Page 7, lines 1 - 11
**S14**	Data analysis	Process by which inferences, themes, etc., were identified and developed, including the researchers involved in data analysis; usually references a specific paradigm or approach; rationale	Page 6, lines 32 – 35<br>Page 7, lines 1 - 11

For peer review only

**S15**	Techniques to enhance trustworthiness	Techniques to enhance trustworthiness and credibility of data analysis (e.g., member checking, audit trail, triangulation); rationale	Page 7, lines 2 – 6, line 7, line 8, lines 15 - 24
**Results/findings**			
**S16**	Synthesis and interpretation	Main findings (e.g., interpretations, inferences, and themes); might include development of a theory or model, or integration with prior research or theory	Page 8, lines 3 - 47<br>Page 9, lines 4 - 46, table 2<br>Page 10, table 3, lines 2 - 23<br>Figure 1 and figure 2
**S17**	Links to empirical data	Evidence (e.g., quotes, field notes, text excerpts, photographs) to substantiate analytic findings	Page 8, lines 21 - 26, lines 40 - 42<br>Page 9, lines 7 – 11
**Discussion**			
**S18**	Integration with prior work, implications, transferability, and contribution(s) to the field	Short summary of main findings; explanation of how findings and conclusions connect to, support, elaborate on, or challenge conclusions of earlier scholarship; discussion of scope of application/generalizability; identification of unique contribution(s) to scholarship in a discipline or field	Page 11, lines 3 – 10, lines 33 – 35, lines 37 - 44<br>Page 12, lines 6 – 8, lines 35 – 43, lines 48 – 50<br>Page 13, lines 1 - 2
**S19**	Limitations	Trustworthiness and limitations of findings	Page 3, lines 5 - 22
**Other**			
**S20**	Conflicts of interest	Potential sources of influence or perceived influence on study conduct and conclusions; how these were managed	Page 21, lines 24 - 29
**S21**	Funding	Sources of funding and other support; role of funders in data collection, interpretation, and reporting	Page 21, lines 18 - 20

For peer review only
